# Acidosis induces RIPK1-dependent death of glioblastoma stem cells via acid-sensing ion channel 1a

**DOI:** 10.1038/s41419-022-05139-3

**Published:** 2022-08-12

**Authors:** Jan Clusmann, Klaus-Daniel Cortés Franco, David Alejandro Corredor Suárez, Istvan Katona, Maria Girbes Minguez, Nina Boersch, Karolos-Philippos Pissas, Jakob Vanek, Yuemin Tian, Stefan Gründer

**Affiliations:** 1 Institute of Physiology, RWTH Aachen University, Aachen, Germany; 2 Institute of Neuropathology, RWTH Aachen University, Aachen, Germany

**Keywords:** Cancer stem cells, Cancer microenvironment, CNS cancer

## Abstract

Eliciting regulated cell death, like necroptosis, is a potential cancer treatment. However, pathways eliciting necroptosis are poorly understood. It has been reported that prolonged activation of acid-sensing ion channel 1a (ASIC1a) induces necroptosis in mouse neurons. Glioblastoma stem cells (GSCs) also express functional ASIC1a, but whether prolonged activation of ASIC1a induces necroptosis in GSCs is unknown. Here we used a tumorsphere formation assay to show that slight acidosis (pH 6.6) induces necrotic cell death in a manner that was sensitive to the necroptosis inhibitor Nec-1 and to the ASIC1a antagonist PcTx1. In addition, genetic knockout of ASIC1a rendered GSCs resistant to acid-induced reduction in tumorsphere formation, while the ASIC1 agonist MitTx1 reduced tumorsphere formation also at neutral pH. Finally, a 20 amino acid fragment of the ASIC1 C-terminus, thought to interact with the necroptosis kinase RIPK1, was sufficient to reduce the formation of tumorspheres. Meanwhile, the genetic knockout of MLKL, the executive protein in the necroptosis cascade, did not prevent a reduction in tumor sphere formation, suggesting that ASIC1a induced an alternative cell death pathway. These findings demonstrate that ASIC1a is a death receptor on GSCs that induces cell death during prolonged acidosis. We propose that this pathway shapes the evolution of a tumor in its acidic microenvironment and that pharmacological activation of ASIC1a might be a potential new strategy in tumor therapy.

## Introduction

Necroptosis is a form of regulated cell death that relies on a cascade involving phosphorylation and activation of serine/threonine receptor-interacting protein kinase 1 (RIPK1) and RIPK3 [1–3]. This leads to oligomerization and translocation of the mixed lineage kinase domain-like pseudokinase (MLKL) to the plasma membrane, which finally elicits membrane rupture [4, 5]. The canonical inhibitor of RIPK1 signaling is necrostatin 1 (Nec-1) [6]. Necroptosis can be induced by tumor-necrosis-factor receptor 1 (TNFR1) signaling [7], but TNFR-independent pathways for necroptosis induction via RIPK1 are less well understood [6, 8, 9].

Recently, it has been shown that necroptosis of neurons can be induced by the activation of acid-sensing ion channel 1a (ASIC1a) [10, 11] independently of TNFR1. ASIC1a is a neuronal Na^+^ channel that is expressed throughout the nervous system [12, 13] and is activated by extracellular protons with high sensitivity (half-maximal activation at pH 6.6) [14]. Physiologically, it contributes to sensing transient decreases in pH as they occur during synaptic transmission. Even though ASIC1a desensitizes within a few seconds, it can also signal sustained acidosis, for example during ischemic stroke. In animal models of ischemic stroke, ASIC1a is responsible for acid-induced cell death: the specific toxin inhibitor psalmotoxin 1 (PcTx1) [15] or knockout of the ASIC1a gene significantly reduces neuronal death [16, 17]. It has recently been proposed that acid-induced cell death depends on proton-induced slow conformational changes of the intracellular ASIC1a N- and C-termini, leading to recruitment of RIPK1 to the ASIC1a C-terminus and to its activation [10, 11]. This would be a completely new way of necroptosis induction. It is unclear whether ASIC1a induces necroptosis also in other circumstances.

Sustained acidosis is also a common feature of the tumor microenvironment (TME) of many cancer types including glioblastoma (GBM) [18, 19], the most common and most malignant brain tumor [20]. GBM has a fast proliferation rate, resulting in a mildly acidic extracellular pH (pH_e_) [21], between 6.6 and 7.2 [19]. Glioblastoma stem cell (GSC) lines are a suitable in vitro model to investigate GBM because they represent the genotype and in vivo biology of human glioblastoma more closely than common serum-cultured cell lines [22–25]. We have previously shown that GSC lines express functional ASIC1a [26], but the function of ASIC1a in GSCs is unknown.

Here, we investigated whether ASIC1a induces cell death in GSCs under acidic conditions. We show that mild acidosis strongly reduced the formation of tumorspheres in an ASIC1a- and Nec-1-dependent fashion in two different GSC lines. A specific ASIC1a activator and a small peptide mimicking the region of the intracellular ASIC1a C-terminus that interacts with RIPK1 similarly reduced sphere formation of GSCs in vitro. In summary, our results show that activation of ASIC1a reduces the formation of GSC spheres by inducing Nec-1-dependent cell death.

## Results

### Mild acidosis strongly reduces the proliferation of GSCs

**Fig. 1:**
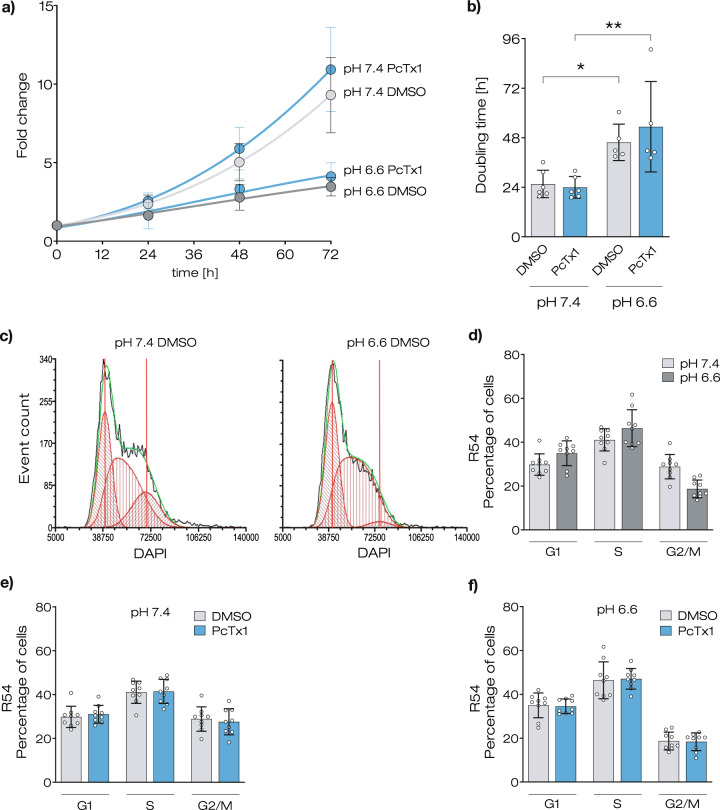
Acidic pH decreases proliferation without effect on cell cycle in R54 GSCs. **a** Growth curves for R54 cells incubated in a medium with neutral (pH 7.4) or acidic pH (pH 6.6) with or without PcTx1. The cells were counted every 24 h for 4 days. Data are shown as mean ± SD, *n* = 6. **b** Doubling time of R54 cells, as determined with the data from panel (**a**). Data are shown as mean ± SD; *p < 0.05; **p < 0.01 (one-way ANOVA followed by Sidak’s post-hoc test). **c** Representative histograms of flow cytometry. **d** Percentage of cells in different phases of the cell cycle at pH 7.4 and at pH 6.6. **e** As in **d** but for pH 7.4 without and with PcTx1. **f** As in **e**, but for pH 6.6. The values for the DMSO condition in **e,** **f** are from panel (**d**). Data are shown as mean ± SD of nine samples from three independent experiments. Statistical analysis by two-way ANOVA with Bonferroni correction.

To assess whether an acidic microenvironment influences the proliferation of GSCs, we determined the proliferation rate of R54 cells, CD133+, pro-neural-like GSCs [27], at neutral and slightly acidic pH (pH 7.4 and 6.6, respectively), by counting cells for 4 consecutive days. To additionally assess whether ASIC1a plays a role in the proliferation of GSCs, part of the cells was incubated with the potent ASIC1a inhibitor PcTx1 (100 nM) [15]. We found that slightly acidic pH reduced the growth strongly (*p* < 0.0001; Fig. 1a), such that the doubling time increased almost two-fold (from 25.6 ± 6.6 h at pH 7.4 to 45.8 ± 8.8 h at pH 6.6; *n* = 6; *p* = 0.049; Fig. 1). PcTx1, however, had no effect on growth rates and doubling time (*p* = 0.800; Fig. 1b), suggesting that the reduced proliferation at acidic pH was independent of ASIC1a.

To assess whether the decreased proliferation rate in an acidic microenvironment was mirrored by a longer duration of specific phases of the cell cycle, we performed FACS analysis of DAPI-labeled R54 cells after 3 days of cultivation at pH 7.4 or at pH 6.6. Typical for rapidly proliferating cells, ~40% of the cells at pH 7.4 were in S phase (Fig. 1c, d). Strikingly, we found the same for cells at pH 6.6. Furthermore, there were only slight changes for the fraction of cells in G_1_ and G_2_/M phases at different pH (Fig. 1d). These results indicate that all phases of the cell cycle were slowed down almost proportionally at acidic pH. Neither incubation with PcTx1 (Fig. 1f) nor the RIPK1-inhibitor Nec-1 (Supplementary Fig. 2a, b) had substantial effects on cell cycle progression at neutral or acidic conditions.

We confirmed these results for the CD133−, mesenchymal-like GSC line R8 [27] (Supplementary Figs. 1 and 2c, d).

### Acidic pH reduces metabolism of R54 cells but does not induce apoptosis or necrosis

**Fig. 2:**
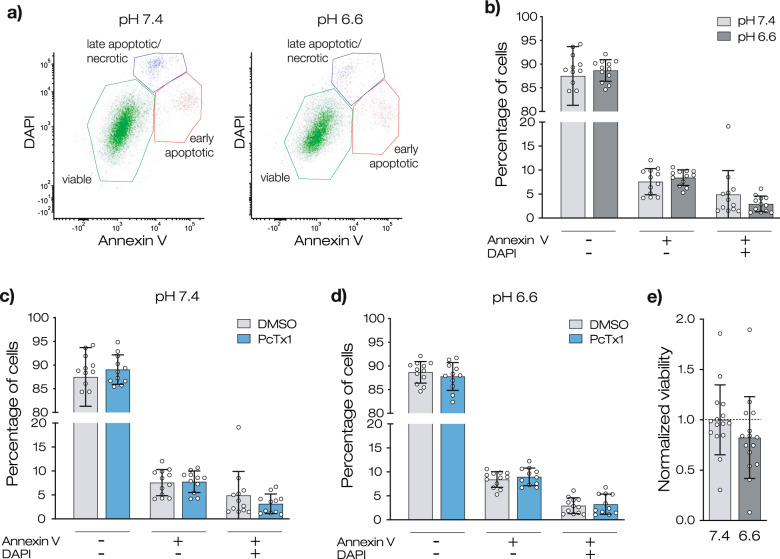
Acidic pH does not induce apoptosis in R54 GSCs. **a** Representative dot plots of flow cytometry measurements. **b** Percentage of viable, early apoptotic, and late apoptotic R54 cells at pH 7.4 and at pH 6.6. **c** As in **b** but for pH 7.4 with and without PcTx1. (**d**) as in **c** but for pH 6.6. The values for the DMSO condition in (**c**, **d**) are from panel (**b**). Data are shown as mean ± SD of nine samples from three independent experiments. Statistical analysis by two-way ANOVA with Bonferroni correction. **e** Normalized viability (mean ± SD) of R54 cells after 24 h incubation at pH 7.4 or at pH 6.6. *n* = 15 from three biological replicates. Statistical analysis by unpaired Student’s *t*-test.

To assess whether the changes in cell growth were caused by an increase in cell death, we used an annexin V/DAPI assay. We found that ~90% of R54 cells were viable (annexin V- and DAPI-negative) and that most of the remaining cells were early apoptotic cells (annexin V-positive/DAPI-negative), irrespective of pH (Fig. 2). Less than 5% of the cells were necrotic and late apoptotic cells (annexin V-positive/DAPI-positive). Thus, acidic pH did not induce apoptosis or necrosis of GSCs under these conditions. To examine whether acidic pH affected the metabolic activity of R54 cells, we performed an alamarBlue Assay (Fig. 2e). Compared with R54 cells grown at pH 7.4, the viability of cells grown at pH 6.6 for 24 h was reduced by 18% (*p* = 0.2131; *n* = 15).

In summary, our results so far revealed a substantially reduced growth of GSCs at acidic pH. This decrease was accompanied by an almost proportional slowing of all phases of the cell cycle and by a slight reduction of cell metabolism. In contrast, so far, our results did not reveal an increase in cell death at acidic conditions.

### Acidic pH reduces tumorsphere formation in a Nec-1-dependent manner

**Fig. 3:**
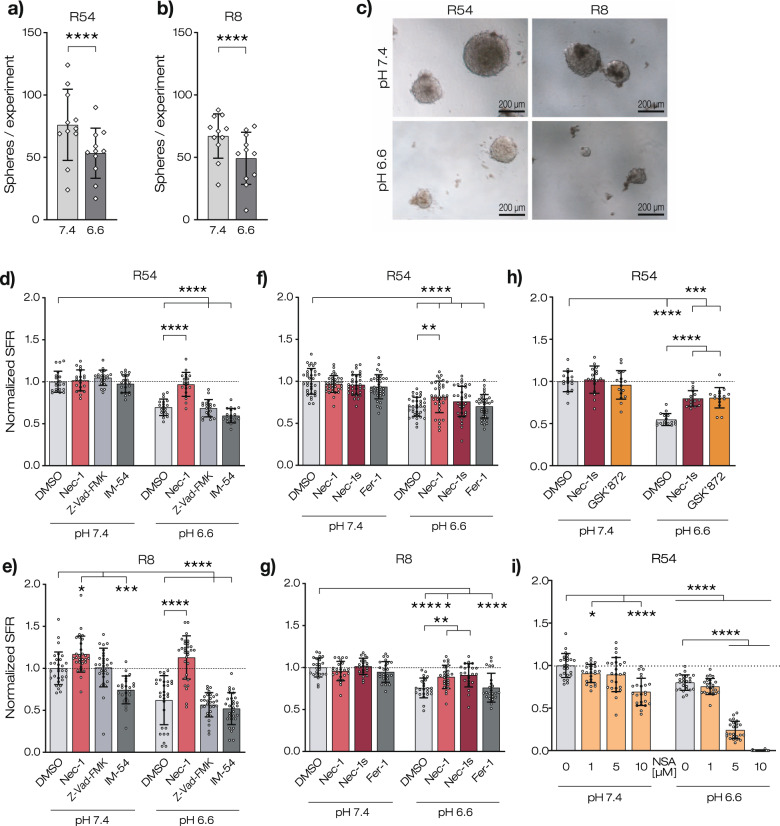
Nec-1 rescues the reduced sphere formation rate at acidic pH. **a**, **b** Sphere numbers (mean ± SD) at pH 7.4 or at pH 6.6 at d12. 1 data point represents 1 experiment with *n* > 8 wells/experiment. **c** Representative images of spheroids after 12 days incubation at pH 7.4 or at pH 6.6. **d**, **e** SFR (mean ± SD) after 12 days incubation at pH 7.4 or at pH 6.6 with 20 µM Nec-1, 20 µM Z-Vad-FMK, or 10 µM IM-54. SFR was normalized to DMSO pH 7.4 control, which was 80.7 ± 24.5 for R54 and 62.2 ± 17.25 for R8. 1 data point represents 1 well. *n* ≥ 19 wells per condition from 2 (**d**, **g**, **i**) or 3 (**e**, **f**) independent experiments. (**f**, **g**) as in (**d**) but with 20 µM Nec-1, 20 µM Nec-1s or 2 µM Fer-1 with a mean of 47.4 ± 6.6 spheres for R54 and 72.4 ± 7.9 spheres for R8. **h** as in **d** but with 20 µM Nec-1s or 10 µM GSK’872 with a mean of 82.2 ± 8.9 spheres. **i** as in **d**, but with different concentrations of NSA, with a mean of 92 ± 12.8 spheres. *p < 0.05; **p < 0.01; ***p < 0.001; ****p < 0.0001 (one-way ANOVA followed by Dunnett’s post-hoc test).

To better mimic physiologically relevant conditions, we next employed a tumorsphere formation assay. A tumorsphere can develop from the proliferation of a single GSC. Thus, the number of tumorspheres formed can be used to estimate the percentage of GSCs in a tumor cell population. To investigate the sphere formation rate (SFR) at neutral and at slightly acidic pH, we seeded 200 individual R54 or R8 cells in a well and cultivated them for 12 days at pH 7.4 or pH 6.6. At pH 7.4, 80.7 ± 24.5 and 62.2 ± 17.3 spheres formed for R54 and R8, respectively (mean ± SD; Fig. 3a, b). Strikingly, at pH 6.6 SFR was significantly reduced by >25% (71.8 ± 6.7% and 63.7 ± 19.5% of control for R54 and R8, respectively; *n* = 11 experiments for R54 and *n* = 11 experiments for R8; *p* < 0.0001) (Fig. 3a, b), suggesting that acidic pH reduces the number of GSCs or their capacity to form spheres. The diameter of the spheres was also ~2-fold reduced (from 211 ± 60 to 126 ± 34 µm for R54 and from 234 ± 63 to 121 ± 26 µm for R8, respectively; *n* = 100, *p* < 0.0001; Supplementary Fig. 3a, c, e), consistent with the reduced growth of GSC lines at acidic pH (Fig. 1).

To investigate whether the reduced SFR at acidic pH might be a result of a reduced number of GSCs due to regulated cell death, we applied three different cell death inhibitors (Fig. 3d, e): Z-Vad-FMK, a pan-caspase inhibitor to inhibit apoptosis [28], IM-54 to inhibit ROS-induced necrosis [29] and necrostatin-1 (Nec-1) to inhibit necroptosis [6]. We found that only Nec-1 had a striking effect on SFR: while resulting in only minor effects at pH 7.4 (2.5% increase for R54, *p* = 0.99, and 16.3% for R8, *p* = 0.05), at pH 6.6, it strongly increased SFR by 27.4% for R54 (*p* < 0.0001) and by 49.3% for R8 (*p* < 0.0001). Nec-1 indeed completely reversed the effect of acidic pH on SFR, such that the SFR at pH 6.6 with Nec-1 was no longer different from the SFR at pH 7.4 (*p* = 0.98 for R54 and *p* = 0.21 for R8).

In contrast to its effects on the SFR, Nec-1 did not affect sphere diameters, which were still reduced at acidic pH (Supplementary Fig. 3a, c), suggesting that once spheres are formed, they grow Nec-1 independently. Similarly, R54 cells incubated with Nec-1 showed no significant effects on cell death in the Annexin V/DAPI assay (Supplementary Fig. 4).

As Nec-1 has also other targets than RIPK1, we investigated the effect of the more specific necroptosis inhibitor 7-Cl-O-Nec-1 (Nec-1s) [30] (Fig. 3f, g). For comparison, Fer-1, a ferroptosis inhibitor [31], was also used. For R8, Nec-1s increased the SFR at pH 6.6 significantly (*p* = 0.0011), comparable to Nec-1. For R54, Nec-1s increased SFR only in two out of three experiments. Therefore, although Nec-1s increased the SFR by 6%, this increase was not significant (*p* = 0.244). However, Nec-1s robustly and significantly increased the SFR in the following experiment (*p* < 0.0001, Fig. 3h). Application of Fer-1 altered the SFR neither at pH 7.4 nor at pH 6.6 for both R54 and R8 (Fig. 3f, g).

In the canonical pathway of necroptosis, activation of RIPK1 is followed by recruitment of RIPK3 and MLKL. A survey of The Cancer Genome Atlas (TCGA) on expression in GBM tissue of RIPK1, RIPK3, and MLKL revealed overexpression of each protein, as well as a correlation between low expression and longer survival of patients (Supplementary Fig. 6). We, therefore, investigated the effect of the RIPK3 inhibitor GSK’872 and the MLK inhibitor necrosulfonamide (NSA) on SFR (Fig. 3h, i). GSK’872 significantly increased the SFR by 25.8% (*n* = 16, *p* < 0.0001), suggesting the involvement of RIPK3 in acid-induced cell death (Fig. 3h). Unexpectedly, however, NSA reproducibly induced cell death in a concentration- and pH-dependent manner (Fig. 3i). Although this effect was unexpected, it had previously been observed [32, 33] and characterized [34] by others.

### Reduced sphere formation rate at pH 6.6 is ASIC1a-dependent

**Fig. 4:**
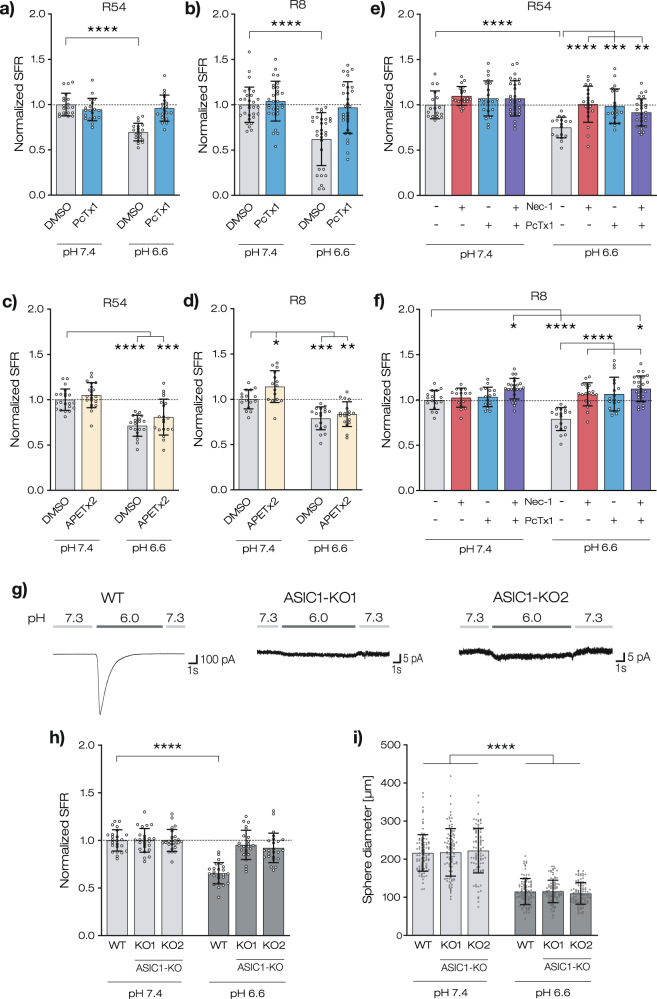
Reduced sphere growth at pH 6.6 is ASIC1a-dependent. **a**–**f** SFR after 12 days at pH 7.4 or at pH 6.6 with 100 nM PcTx1, 500 nM APETx2, or 100 nM PcTx1 and 20 µM Nec-1 combined. SFR was normalized to DMSO pH 7.4 control, which was 74.5 ± 11.1 for **a**, 46.3 ± 6.7 for **b**, 102.4 ± 11.5 for **c** and **e**, and 77 ± 7.9 for **d** and **f**. 1 data point represents 1 well. *n* ≥ 17 wells per condition, 2 independent experiments. *p < 0.05; **p < 0.01; ***p < 0.001; ****p < 0.0001. One-way ANOVA was followed by Dunnett’s post-hoc test. **g** Traces of whole-cell patch clamp experiments with wildtype or ASIC1a knockout R54 cells. ASIC currents were elicited by a pH drop from pH 7.3 to 6.0. Each trace is representative of five similar measurements. **h** SFR after 12 days at pH 7.4 or at pH 6.6. SFR was normalized to the respective pH 7.4 control, which was 104.7 ± 11 for wt R54, 77 ± 9.1 for KO1, and 60.4 ± 6.8 for KO2. *n* = 24 wells per condition from 2 independent experiments. **i** Sphere diameters (mean ± SD) in µm of 100 spheres per condition after 12 days at pH 7.4 or at pH 6.6. Measurements are from 2 independent experiments. Data points represent single sphere diameters. *p < 0.05; **p < 0.01; ***p < 0.001; ****p < 0.0001 (one-way ANOVA followed by Sidak’s post-hoc test).

Because pH 6.6 can activate ASICs [14], we investigated the effects of PcTx1 and the ASIC3 inhibitor APETx2 [35] on SFR (Fig. 4a–d). We found that for both R54 and R8, PcTx1 significantly increased SFR at pH 6.6 by >25% (by 26.5% for R54 and by 34.8% for R8; *n* = 19–20 for R54 and *n* = 29 for R8; *p* < 0.0001), while it did not affect SFR at pH 7.4. This increase in SFR by PcTx1 was very similar to the increase by Nec-1. In contrast, APETx2 had no effects on the SFR at acidic pH (*p* = 0.114 for R54, *p* = 0.779 for R8). Co-application of PcTx1 and Nec-1 resulted in a similar increase in SFR as an application of either Nec-1 or PcTx1 alone, suggesting that both substances interfere with the same pathway (Fig. 4e, f). Like Nec-1, PcTx1 did not increase the diameter of spheres (Supplementary Fig. 3b, d), suggesting that once formed, spheres grow independently of ASIC1a, consistent with the results showing that PcTx1 did not affect growth rate at acidic pH (Fig. 1b).

To corroborate the involvement of ASIC1a in reduced SFR at acidic pH, we performed a knockout of the *ASIC1a* gene via the CRISPR/Cas9 method in R54. Electrophysiological characterization via whole-cell patch-clamp showed that the typical ASIC1 inward current at acidic pH [26] could not be elicited in two independent knockout lines (KO1 and KO2) (Fig. 4g). For WT R54, SFR was reduced to 65.4 ± 10.8% at pH 6.6 (*n* = 24, *p* < 0.0001; Fig. 4h), consistent with the previous experiments. Strikingly, however, in the two knockout cell lines, there was no significant reduction of the SFR at acidic pH (95.2 ± 15.0% for KO1, *n* = 24, *p* = 0.492 and 92.2 ± 13.9% for KO2, *n* = 24, *p* = 0.110), confirming that the reduction in SFR was ASIC1a-dependent. In contrast, the ASIC1a knockout did not affect sphere sizes (Fig. 4i).

### Activation of ASIC1a can reduce sphere formation independent of pH

**Fig. 5:**
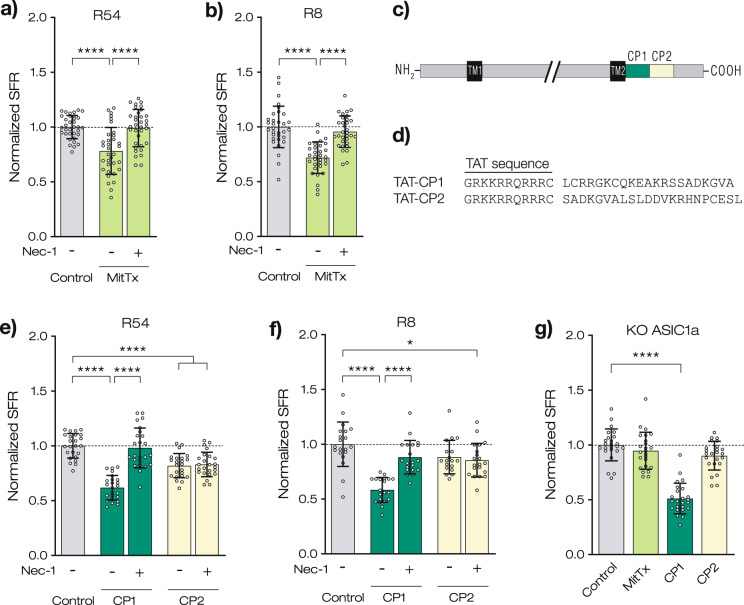
Sphere growth can be reduced by MitTx or a peptide resembling the C-Terminus of ASIC1a. **a**, **b** SFR after 7 days at pH 7.4, without or with 20 nM MitTx. SFR was normalized to pH 7.4 control, which was 100.4 ± 10.5 for R54 and 79.1 ± 11.8 for R8. **c** Schematic illustration of the ASIC1a protein with N- and C-Termini, transmembrane domains (TM1/2) (extracellular loop shortened) and location of CP1 and CP2 illustrated in color. **d** Sequence of peptides CP1 and CP2, combined with HI-viral peptide transduction sequence TAT. **e**, **f** SFR after 7 days at pH 7.4, with or without 10 µM CP1 or 10 µM CP2 and with or without 20 µM Nec-1. SFR was normalized to pH 7.4 control, which was 99.9 ± 11.0 for R54 and 82.2 ± 11.6 for R8. **g** SFR of ASIC1a KO cells after 7 days at pH 7.4 without or with 20 nM MitTx, 10 µM CP1, or 10 µM CP2. SFR was normalized to pH 7.4 control, which was 77.1 ± 9.5 **a**, **b**, **e**–**g** *n* ≥ 17 wells per condition from 2 independent experiments. Mean ± SD are shown. *p < 0.05; ****p < 0.0001 (one-way ANOVA followed by Dunnett’s post-hoc test).

To further corroborate the role of ASIC1a in the induction of Nec-1-dependent cell death, we asked whether the snake toxin MitTx, a specific and highly potent ASIC1 agonist [36], can reduce SFR even at neutral pH. We found that application of MitTx indeed strongly reduced the SFR at pH 7.4 to 76.9 ± 12.1% of control (*p* < 0.0001) for R54 and to 73.1 ± 10.3% of control (*p* < 0.0001) for R8 (Fig. 5a, b). In contrast, co-application of Nec-1 with MitTx completely prevented the reduction in SFR (97.9 ± 9.7%, *p* = 0.961 for R54 and 96.3 ± 10.9%, *p* = 0.622 for R8).

To test more directly for induction of cell death by ASIC1a, we tested the relevance of the ASIC1a C-terminus. It has recently been shown that a peptide from the ASIC1a C-terminus is sufficient to induce cell death in mouse neurons [10]. We applied the corresponding synthetic peptide of the human ASIC1a C-terminus, fused to the cell-penetrating peptide TAT of HIV [37], in the tumorsphere formation assay (Fig. 5c–f). We found that the active peptide, CP1, robustly reduced the SFR at pH 7.4 to 61.8 ± 10.4% for R54 cells (*p* < 0.0001) and to 57.9 ± 10.3% for R8 cells (*p* < 0.0001)—even stronger than MitTx. This reduction was abolished when Nec-1 was co-applied. A control peptide from the ASIC1a C-terminus, CP2, decreased SFR to 81.9 ± 10.6% for R54 (*p* < 0.0001) and to 87.4 ± 10.7% for R8 (*p* = 0.08), but this decrease was not reversed by Nec-1. We attribute this effect of CP2 to a toxic effect of the TAT peptide, which has previously been observed by others [10, 11].

MitTx slightly reduced the diameter of R8 but not of R54 spheres (Supplementary Fig. 5a, c). This reduction was not Nec-1 dependent, however, suggesting that it was independent of the pathway responsible for the reduced SFR. CP1 also reduced sphere diameter for both R54 and R8 (*p* < 0.0001) but this reduction was partially reversed by Nec-1 (Supplementary Fig. 5b, d). CP2 increased sphere diameter in the absence of Nec-1 (Supplementary Fig. 5b, d), a finding for which we have no explanation yet.

Knocking out ASIC1 in R54 cells prevented the decrease in SFR by MitTx (94.7 ± 16.2%, *p* = 0.464). This was expected, as MitTx binds to the extracellular domain of ASIC1 [38] and should therefore not bind to ASIC1 KO cells. In contrast, CP1 decreased the SFR in ASIC1 KO cells to 51.07 ± 11.08% (*p* < 0.0001), suggesting that CP1 is sufficient to induce cell death in the absence of ASIC1 (Fig. 5g).

### Acidic pH induces morphological hallmarks of necrosis at an early phase but not a late phase of sphere growth

**Fig. 6:**
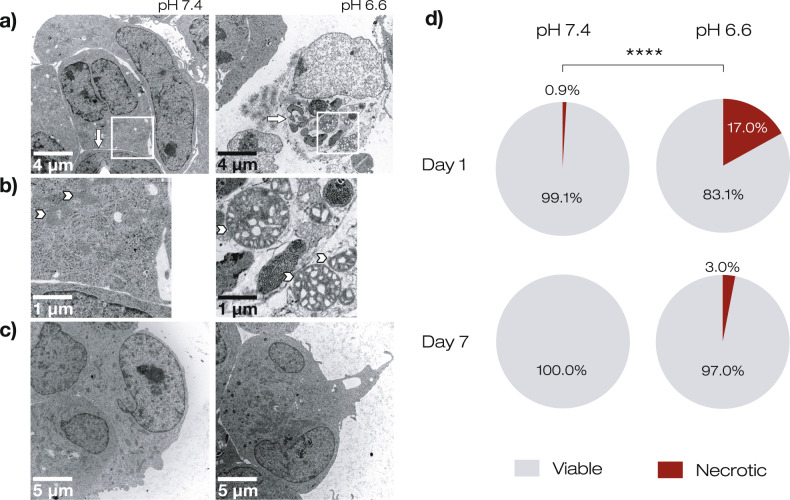
Transmission electron microscopy of R54 GSCs reveals necrosis at acidic pH early during sphere formation. **a**, **b** Representative TEM images of GSCs maintained for 1 day at pH 7.4 or at pH 6.6. White squares in **a** indicate regions that are shown on an expanded scale in (**b**). Arrows indicate plasma membrane, arrowheads mitochondria. **c** Representative TEM images of GSCs maintained for 7 days at pH 7.4 or at pH 6.6. **d** Pie charts indicating the percentage of cells with normal vs. necrotic morphology in TEM after 1 day or after 7 days at pH 7.4 or at pH 6.6. At d1, *n* = 106 for pH 7.4 and *n* = 118 for pH 6.6, respectively. At d7, *n* = 107 for pH 7.4 and *n* = 132 for pH 6.6, respectively. ****p < 0.0001 (Fisher’s exact test).

To assess cell death more directly in GBM tumorspheres, we examined the morphology of R54 cells using transmission electron microscopy (TEM) 1 day (Fig. 6a, b, d) and 7 days (Fig. 6c, d) after sphere formation. At d1 and pH 7.4, 105 of 106 GSCs showed normal cellular morphology. Interestingly, however, at d1 and pH 6.6, 20 of 118 cells displayed a typical necrotic morphology, including plasma membrane rupture and organelle swelling (*p* < 0.0001, Fisher’s exact test). In contrast, at d7 and pH 6.6, GSCs showed no obvious apoptotic or necrotic morphology (*p* = 0.130, Fisher’s exact test; Fig. 6c); the same was found for cells at pH 7.4. This finding concurs with the interpretation that acidosis induces necrotic cell death early during the formation of tumorspheres, but not after one week of acidosis.

### Acidosis or ASIC1 activation induces phosphorylation of RIPK1 in R54 GSCs

RIPK1 contains an N-terminal kinase domain and is phosphorylated at multiple positions. Specifically, phosphorylation of RIPK1 on serine 166 (S166) is crucial for its activation [1] and an initial step in the necroptosis pathway. To test whether RIPK1 is present in R54 GSCs and whether acidosis induces phosphorylation of RIPK1, we assessed the abundance and phosphorylation of RIPK1 on S166 in R54 cells by Western blotting. Spheres were maintained at pH 6.6 for different periods of time (0.5, 1, 2, or 6 h), lysed and proteins were separated by PAGE. For the 6 h time point, we also added Nec-1s or PcTx1 to inhibit RIPK1 or ASIC1, respectively. Additionally, we applied MitTx at pH 7.4 to pharmacologically activate ASIC1. As a positive control, we induced necroptosis at pH 7.4 by tumor necrosis factor α (TNF-α) in combination with Z-VAD-FMK to inhibit apoptosis and the SMAC mimetic BV6 to inhibit the NF-kB survival pathway (T/S/Z) [39].

**Fig. 7:**
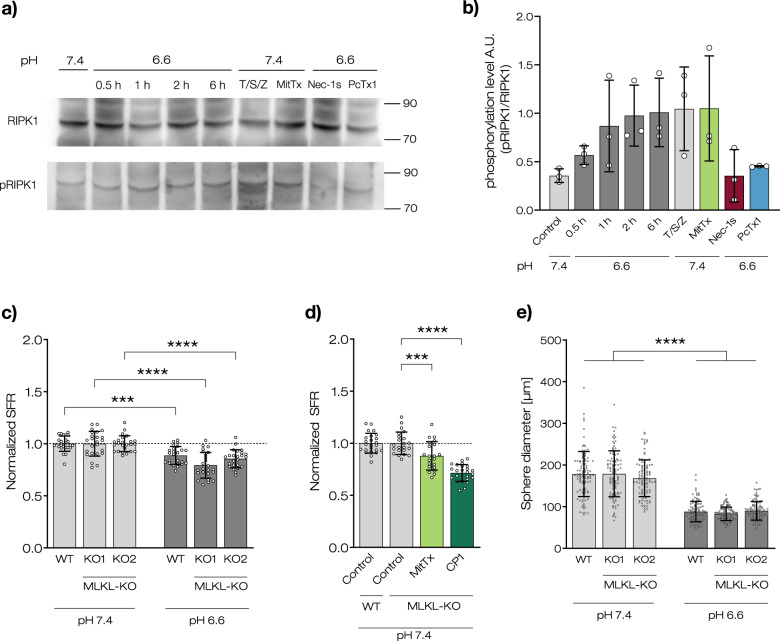
Acidic pH and ASIC1a-activation induce phosphorylation of RIPK1 in R54 GSCs. **a** Western blots of RIPK1 and pRIPK1 from R54 GSCs, incubated either at pH 7.4 or at pH 6.6 for different periods of time (0.5, 1, 2, or 6 h) with or without different inhibitors and activators as indicated. T/S/Z = TNF-α, BV6 and z-Vad-FMK. 20 µM Nec-1s or 100 nM PcTx1 were applied at acidic pH for 6 h. **b** Densitometric quantification of western blot bands for pRIPK1 normalized to RIPK1. Data are shown as mean ± SD and are from three western blots from three independent experiments. **c** SFR for wt or MLKL-KO cells after 11 days at pH 7.4 or at pH 6.6. SFR was normalized to the respective pH 7.4 control, which was 81.5 ± 5.7 for wt, 71.3 ± 8.2 for KO1, and 74.8 ± 5.5 for KO2. 1 data point represents 1 well. *n* = 24 wells per condition, three independent experiments. **d** SFA for KO2 as in **c**, but without or with 20 nM MitTx or 10 µM CP1. SFR was normalized to the respective pH 7.4 control, which was 84.1 ± 7.8 for wt and 63.2 ± 6.2 for KO2. **e** Sphere diameters (mean ± SD) in µm of 100 spheres per condition after 11 days at pH 7.4 or at pH 6.6. Measurements are from two independent experiments. Data points represent single sphere diameters. ***p < 0.001; ****p < 0.0001 (one-way ANOVA followed by Sidak’s post-hoc test).

Western blotting revealed the presence of RIPK1 in R54 GSCs (Fig. 7a). While T/S/Z-treatment did not change RIPK1 abundance, it strongly increased the abundance of phospho-RIPK1. Likewise, treatment with pH 6.6 strongly increased the abundance of phospho-RIPK1 in a time-dependent manner, suggesting that acidic pH, like TNF-α signaling, induces phosphorylation of RIPK1 (Fig. 7a, b). While the increase in phospho-RIPK1 was prevented by the application of Nec-1s or PcTx1, MitTx increased the abundance of phospho-RIPK1 also at neutral pH. These data indicate that ASIC1 activation induces phosphorylation and activation of RIPK1.

So far our results suggest that ASIC1a activation leads to necroptosis in R54 GSCs. MLKL is the executioner protein in the necroptosis cascade, downstream of RIPK1 and RIPK3. We created two independent CRISPR/Cas9 knockouts of the *MLKL* gene to investigate whether the reduced SFR was indeed due to necroptosis. Surprisingly and in contrast to the ASIC1 KO, knocking out MLKL did not prevent the decreased SFR at acidic pH. For KO1, the SFR decreased to 79.3 ± 12% (*p* < 0.0001) at acidic pH, for KO2 to 85.6 ± 7.7% (*p* < 0.0001) (Fig. 7c). Furthermore, both MitTx and CP1 still reduced SFR even at neutral pH in MLKL KO cells (87.3 ± 8.3%, *p* = 0.0009 for MitTx, 71.5 ± 6.4%, *p* < 0.0001 for CP1) (Fig. 7d). Meanwhile, the sphere diameter was not affected by the knockout of the *MLKL* gene (Fig. 7e). Collectively, these results suggest that the ASIC1-mediated reduction in SFR was, despite the involvement of RIPK1, independent of MLKL.

## Discussion

Our results consistently show that activation of ASIC1a by slight acidosis reduces the SFR of the GSCs R54 and R8 in a Nec-1-dependent manner. The evidence for this conclusion is severalfold: first, the reduction in SFR at pH 6.6 was rescued by Nec-1, its more specific analog Nec-1s [30], and the potent ASIC1a inhibitor PcTx1. Second, acidic pH did not reduce SFR in R54 cells with a genetic knock-out of ASIC1a. Third, MitTx, a potent and specific toxin agonist of ASIC1a, reduced SFR at neutral pH. And fourth, a peptide from the C-terminus of ASIC1a was able to reduce SFR at neutral pH, in ASIC1a WT—as well as in ASIC1a KO cells. Together, these results provide compelling evidence that ASIC1a is a novel death receptor in patient-derived GSCs.

Sensitivity of reduced SFR to the RIPK1 inhibitors Nec-1 and Nec-1s and to the RIPK3 inhibitor GSK’872 and necrotic morphology of R54 GSCs after 1 day of mild acidosis suggested that the reduced SFR was due to necroptosis. However, MLKL knockout experiments revealed that the execution of ASIC1-mediated cell death did not depend on MLKL and therefore was not necroptosis. Because the reduced SFR was also not sensitive to Z-Vad-FMK, the cell death was also not apoptosis. Thus, it appears that the ASIC1a-mediated reduction in SFR relied on a new cell death pathway that clearly involved RIPK1 and possibly also RIPK3. Details regarding the downstream cascade have to be further elucidated in the future.

Strikingly, once tumorspheres were formed, there was no further evidence for induction of cell death by acidosis: PcTx1 did not reduce the doubling time at pH 6.6, acidosis did not increase the number of early apoptotic or late apoptotic/necrotic cells in an annexin V/DAPI assay, Nec-1 did not increase sphere diameter at pH 6.6, and after 7 days of mild acidosis, cells no longer had necrotic morphology. Thus, these results indicate that induction of cell death via acidosis/activation of ASIC1a and a Nec-1-dependent pathway is an early event. At present, it is not clear whether GSCs become resistant to acidosis after prolonged incubation or whether only individual cells but not cells in spheres are vulnerable to acidosis/activation of ASIC1a. In any case, the early nature of cell death by acidosis/ASIC1a is reminiscent of a previous study reporting that pretreatment with Nec-1 or Nec-1s was necessary to rescue neurons from acid-induced cell death, suggesting a role for RIPK1 at the onset of acidotoxicity in neurons [10].

It was unexpected that mild acidosis did not induce G1 arrest of GSCs, but rather slowed down all phases of the cell cycle proportionally, leading to a two-fold increase in doubling time. In stark contrast to reduced SFR, the reduced proliferation rate and smaller sphere size at mild acidosis were insensitive to PcTx1 and Nec-1, indicating that these effects are independent of ASIC1a. Reduced proliferation of GSCs at acidic pH had already previously been shown [27]. In contrast, a recent study reported that in two serum-cultured glioblastoma cell lines, A172 and U78MG cells, a slighter acidosis of pH 7.0 did not reduce proliferation [40].

Our results revealed the presence of RIPK1, in particular of RIPK1 phosphorylated at S166, in R54 GSCs. The abundance of phospho-RIPK1 increased after 0.5–6 h of mild acidosis, and this increase was prevented by the RIPK1-inhibitor Nec-1 or the ASIC1a antagonist PcTx1. The abundance of phospho-RIPK1 also increased after ASIC1 activation by MitTx at neutral pH. Together, these results support the idea that slight acidosis and consecutive ASIC1 activation induced RIPK1-phosphorylation. Strikingly, however, MLKL knock-out did not prevent reduction in SFR by slight acidosis, strongly arguing against the involvement of the canonical necroptosis pathway that depends on MLKL. Future studies need to unravel the cell death pathway that is induced by mild acidosis in GSCs.

While it is conceivable that cell death induction by acidic TME shapes the evolution of tumor cells, it remains unclear what the precise impact of the ASIC1a/RIPK1-dependent cell death pathway would be for a tumor in situ. Although brain cancer grows in an acidic TME [19], the pH will fall gradually in tumor tissue, and it is unclear whether this would induce RIPK1-dependent cell death via activation of ASIC1a. Furthermore, the role of pro-inflammatory cell death pathways in cancer therapy is ambiguous [41, 42] and has yet to be characterized for the pathway described here. Similar pathways, such as necroptosis, can activate oncogenic responses by inducing inflammation [43]. On the other hand, they can serve as alternative drug targets to the apoptosis pathway, especially as the release of antigens during cell death could render the tumor more susceptible to immunotherapy [44, 45] and strategies for immune induction are desperately needed [46, 47]. The TGCA data shown in Supplementary Fig. 6 confirm several studies which show increased expression of RIPK1, RIPK3, and MLKL in GBM compared to non-tumor tissue, as well as an unfavorable role for higher expression [48–50]. On the other hand, and in agreement with an anti-tumorigenic role of ASIC1a, the expression of ASIC1a is associated with increased survival time in lower-grade glioma patients [26]. Because ASIC1a activation leads to increased cell death only early during sphere formation, in vivo studies are necessary to reveal whether this pathway is indeed anti-tumorigenic.

GBM is heterogeneous [51]. We, therefore, included two GSC lines from primary GBM with different transcriptional profiles: R54, with a pro-neural-like profile, and R8, with a mesenchymal-like profile [52]. It was remarkable that mild acidosis similarly reduced SFR for both GSC lines in a PcTx1 and Nec-1-dependent manner, suggesting that cell death induction by activation of ASIC1a is common to different GSCs. Sustained acidosis is not only a hallmark of the TME but accompanies many inflammatory and neurodegenerative diseases of the CNS, such as multiple sclerosis (MS), Alzheimer’s (AD), and Parkinson’s disease (PD) as well as traumatic brain injury. ASIC1a activity, causing cell death and inflammation, is increasingly associated with these diseases [53–55] and ASIC1a inhibition ameliorates cell damage [16, 17, 56, 57]. Similarly, yet better characterized, necroptosis has been shown to be a promising drug target in PD, AD, and stroke [58], with RIPK1 inhibition alleviating brain damage in models of MS [59], AD [60, 61], PD [62, 63], and stroke [64, 65]. Because ASIC1a signaling and RIPK1-dependent cell death had so far only been linked to ischemic stroke [10, 11], future studies should investigate the potential role of ASIC1a as an upstream target for inducing RIPK1-dependent cell death in other CNS diseases.

In summary, we show that activation of ASIC1a by mild acidosis reduces the formation of GBM tumorspheres in a RIPK1-dependent manner. This finding might increase our understanding of GBM evolution and might hold therapeutic potential for the treatment of GBM.

## Materials and methods

### Cultivation of GSCs

GSC lines R54 and R8 were established in 2007 [52] and kindly provided by Christoph Beier (Department of Neurology, Odense, Denmark). Both cell lines are wild-type for isocitrate-dehydrogenase (IDH) 1 and 2 [66]. They were regularly checked for mycoplasma contamination using qPCR. Low passages of cells were maintained as spheroids in suspension at 37 °C in a humidified atmosphere with 5% CO_2_. Serum-free medium (DMEM-F12), which contained 1.2 g/l NaHCO_3_, supplemented with 2% B27 supplement, 1% glutamine, 1% MEM vitamin solution, 0.1% epidermal growth factor (EGF), and 0.1% fibroblast growth factor (FGF) with pH 7.4 was used for culture and experiments. For a medium with pH 6.6, DMEM-F12 without NaHCO_3_ was used and 0.4002 g/l NaHCO_3_ was added. The pH of the media under experimental conditions (37 °C, 5% CO_2_) was controlled in agreement with recently proposed guidelines [67] and regularly controlled with a pH meter (827 pH lab; Metrohm, Filderstadt, Germany). pH was titrated by the concentration of NaHCO_3_ and measured before experimental procedures. In addition, substantial acidification of the medium during sphere formation experiments was excluded by observing the color of the medium. Spheroids were trypsinized once per week into individual cells, from which new spheres were generated.

### Growth curves and determination of doubling time

Cells were seeded in 3 ml at a density of 25,000 cells/ml in six-well-plates. They were incubated in four different conditions: at pH 7.4 or at pH 6.6 either with 1:1000 DMSO or with 100 nM PcTx1. For 4 consecutive days after seeding the cells, a 500 μl sample was taken from each well every 24 h. Samples were trypsinized with 0.05% trypsin/HBSS (Thermo Fisher Scientific, Schwerte, Germany) to separate spheres into single cells and the number of cells was determined using a CASY cell counter (Roche, Basel, Switzerland) according to the operator’s manual. The sample from d1 was taken as 0 h. The data were fitted to Gompertz growth functions using the method of least squares and the fit of each data set was compared pairwise with an extra sum of squares F test (Prism, GraphPad Software, San Diego, CA, USA).

The doubling time (*t*_c_) of the cells was calculated using the following equation:

$$A\left( t \right) = A_0 \cdot 2^{\frac{t}{{t_{\rm {c}}}}}$$

*A*_0_ was defined as the cell density at 48 h after seeding the cells and *A* as the cell density at 96 h after seeding the cells; *t* = 48 h.

### Cell cycle analysis

After an incubation period of 3 days under the desired conditions (pH 7.4 or 6.6, each either with 20 μM Nec-1 or with 50–100 nM PcTx1; control was 1:1000 DMSO), GSCs were labeled with DAPI, to quantify the DNA content of the cells. Before harvesting and DAPI-labeling, cells were additionally pulsed with EdU (10 µM) for 3 h (R8 cells) or for 12 h (R54 cells); EdU was fluorescently labeled using the Click-iT™ Plus EdU Alexa Fluor™ 647 Flow Cytometry Assay Kit (Thermo Fisher Scientific).

After the EdU pulse, cells were harvested and incubated in 200 μl 0.05% trypsin/HBSS for 2 min to separate any spheroids and cell aggregates into single cells. Cells were then washed in buffered salt solution (DPBS; PAN Biotech) containing 1% BSA and fixed in 4% paraformaldehyde (PFA) for 15 min at RT. Cells were again washed in DPBS-1% BSA and then permeabilized by resuspending them in 100 μl 1X Click-iT saponin-based permeabilization and wash reagent (Thermo Fisher Scientific). Afterward, a batch of Click-iT™ Plus reaction cocktail containing Alexa Fluor™ 647 (Thermo Fisher Scientific) was prepared, 100 µl were added to each sample and incubated for 40 min at RT, protected from light. Cells were washed twice with the 1X Click-iT saponin-based permeabilization and wash reagent and then resuspended in 200 µl of the same reagent. Finally, 1.25 µl DAPI (2 mg/ml) was added to each sample. Each sample was then filtered while being transferred to flow cytometry tubes and measured using flow cytometry. For R54 cells, only the labeling with DAPI was used for the analysis because with the longer EdU pulse, other cells than those in the S phase were EdU positive.

### Apoptosis assay

To assess apoptosis, cells were labeled using annexin V and DAPI. Cells were maintained for 3 days at the desired conditions (pH 7.4 or 6.6, each either with 20 μM Nec-1 or with 50–100 nM PcTx1; control was 1:1000 DMSO). Afterward, they were harvested and incubated in 100 µl of 0.05% trypsin/HBSS for 1 min at RT to separate spheres into single cells. The trypsin was then diluted with 1 ml DPBS–1% BSA. Cells were spun down and resuspended in 200 µl Annexin V binding buffer (BioLegend). 2 µl Annexin V-APC (BioLegend) and 1 µl DAPI solution (20 µg/ml) were added to each sample. The cells were incubated for 30 min and were then filtered while being transferred to flow cytometry tubes.

### Flow cytometry

Samples were analyzed using a FACSCanto II Cell Analyzer (BD Biosciences, CA, USA). For excitation and detection of DAPI, a violet laser (405 nm) and a 450/40 filter were used, for APC and Alexa Fluor 647, a red laser (640 nm) and a 670/14 filter were used. Data were analyzed using FCS Express 7 (De Novo Software, Pasadena, CA, USA).

### AlamarBlue Assay

1000 cells/well were seeded into a 96-well plate in a final volume of 100 μl culture medium of the desired pH. Cells were incubated for 24 h at pH 7.4 or pH 6.6. After incubation, the medium was replaced by 100 μl fresh culture medium with pH 7.4. 10 μl of alamarBlue HS Viability reagent (Thermo Fisher Scientific) was added to each well and cells were incubated for 2 h. Cell viability was measured through fluorescence intensities (excitation, 560 nm; emission: 590 nm) using the Tecan Infinite M200 plate reader. The assay was repeated three times. The data was analyzed using Microsoft Excel and normalized to the corresponding mean of the pH 7.4 control.

### Sphere formation assay

Spheroids were trypsinized and viable cells were counted by Trypan Blue staining. Cells were seeded at 1 cell/µl and preincubated with test substances, which were dissolved in water (MitTx, CP1, and CP2) or DMSO (else), for 30 min at pH 7.4. A solution containing the solvent (DMSO 1:1000) or water 1:1000 served as control, depending on the utilized solvent. The medium was replaced according to condition and cells were seeded in uncoated 96-well plates with 200 µl per well and *n* = 8–12 wells per condition. Plates were cultured at 37 °C with 5% CO_2_ without medium exchange to allow for undisturbed tumorsphere formation. Tumorspheres were solid, round clusters between 50 and 250 µm in diameter. Tumorspheres per well were counted manually after the indicated time. All experiments were performed with 2–3 biological replicates. Data points in bar graphs each represent 1 well. The experimenter was blinded for the condition. Sphere diameters were determined with IC Measure.

### Generation of the ASIC1 and MLKL knock-out cell lines

For CRISPR-Cas, the pSpCas9(BB)-2A-GFP (PX458) vector (Addgene plasmid # 48138) was purchased from Addgene (Watertown, MA, USA) and linearized with BbsI restriction endonuclease (New England Biolabs; Ipswich, MA, USA). Double-stranded DNA guide sequences, targeting the second coding exon of human ASIC1 (5’-TGTCACCAAGCTCGACGAGG-3’), or targeting different regions of the second coding exon of human MLKL (5’-CACACCGTTTGTGGATGACC-3’ and 5’-TACTCTTCAAGGACGTGAAC-3’), were ligated into PX458 using T4 DNA ligase (Thermo Fisher Scientific). R54 cells were transfected with the resulting plasmids, using Lipofectamine 2000 Transfection Reagent (Thermo Fisher Scientific). After 48 h, cells were sorted using a BD FACSAria III cell sorter (BD Biosciences) using GFP fluorescence. Single clones were expanded for 2 weeks and potential candidates were screened by SYBR Green qPCR and melting curve analysis. Clones with a shift of the melting curve peak of >2 °C were TOPO-cloned into the pCR2.1 vector (Thermo Fisher Scientific). Genetic knockout was confirmed by sequencing of genomic DNA (Supplemental Tables 1 and 2). The absence of functional ASIC1a was additionally confirmed by patch clamp analysis.

### Western blot

At pH 7.4, cells were left untreated as control, and alternatively incubated with 20 nM MitTx for 6 h, or treated with TNF–α (20 ng/ml) and SMAC mimetic BV-6 (10 μM) for 6 h after pretreatment with Z-Vad FMK (20 µM) for 30 min for necroptosis induction. The pH 6.6 group was incubated with acidic pH for 0.5–6 h or alternatively pretreated with 20 µM Nec-1s or 100 nM PcTx1 in pH 7.4 for 30 min followed by acidic pH for 6 h. R54 cells were harvested and lysed on ice for 10 min using RIPA buffer with protease inhibitors (cOmplete Protease Inhibitor Cocktail, Sigma-Aldrich), and phosphatase inhibitor cocktail III (Sigma-Aldrich) to avoid dephosphorylation. The lysed cells and lysis buffer were retrieved, briefly vortexed, and centrifuged for 10 min at 10,000 × *g* at 4 °C. The supernatants were retrieved, and the protein concentration of each sample was quantified using a Bincichoninic acid assay (Thermo-Scientific) in order to load equal amounts of protein. The samples were diluted to a concentration of 5 mg/ml with SDS–PAGE loading buffer (50 mM Tris–Cl pH 6.8, 2% SDS, 10% glycerol, 100 mM DTT, and 0.001% bromophenol blue dye). Samples were separated using SDS–PAGE (10%). Proteins were transferred to PVDF membranes (Roche, Mannheim, Germany), and membranes were blocked for 1 h at RT in 4% BSA in TBS-T (137 mM NaCl, 2.7 mM KCl, 25 mM Tris, 0.1% Tween-20), and probed overnight at 4 °C with primary rabbit monoclonal anti-phospho-RIP1 (Ser166) (1:500, Cell Signaling, #65746), followed by secondary HRP-conjugated anti-rabbit (1:10,000, Invitrogen, #31460) in 4% BSA in TBS-T for 1 h at RT. The membranes were imaged using SuperSignal West Pico Chemiluminescent Substrate (Thermo Fisher Scientific). Antibodies were stripped from the membrane by mild stripping using incubation with stripping buffer (1.5% (w/v) glycine, 0.1% (w/v) SDS, 1% Tween 20, pH 2.2) twice for 10 min, followed by three 10 min washing steps with PBS, and finally washed twice for 5 min using TBS-T. The membrane was then blocked using 4% BSA in TBS-T, and incubated with primary antibody mouse monoclonal anti-RIP1 (1:1000, BD Biosciences, #551042) overnight at 4 °C, followed by secondary HRP-conjugated anti-mouse antibody (1:10,000, Invitrogen, #G21040), and imaged using and SuperSignal West Pico Chemiluminescent Substrate. Blots were imaged using a chemiluminescent imager (Vilber Lourmat, Eberhardzell, Germany). Data were analyzed with ImageJ. For the densitometric analysis, bands in the range of 70–90 kDa were quantified for pRIPK1 and normalized to the density of bands in the same range for RIPK1.

### Transmission electron microscopy

GSCs were cultured at pH 7.4 or pH 6.6 for 1 or 7 days. Cells were collected by centrifugation, washed in DPBS, and immediately fixed with 2.5% glutaraldehyde in 0.1 M phosphate buffer for 24 h, followed by washing in the buffer for a further 24 h. Cell pellets were collected by centrifugation (1000 rpm, 5 min) and embedded in 2% low gelling temperature agarose (#A9414, Sigma). Small blocks of embedded cells were sliced and post-fixed in 2.5% glutaraldehyde for 24 h followed by washing in 0.1 M phosphate buffer for 24 h. Agarose blocks were then incubated in 1% OsO_4_ (in 0.2 M phosphate buffer) for 3 h, washed twice in distilled water, and dehydrated using ascending alcohol concentrations (i.e., 25%, 35%, 50%, 70%, 85%, 95%, and 100%; each step for 5 min). Dehydrated blocks were incubated in propylene oxide followed by subsequent 20 min of incubation in a 1:1 mixture of epon (47.5% glycidether, 26.5% dodenylsuccinic acid anhydride, 24.5% methylnadic anhydride, and 1.5% Tris (dimethyl aminomethyl) phenol) and propylene oxide. The samples were then incubated in epoxy resin for 1 h at room temperature followed by polymerization (28 °C for 8 h, 80 °C for 2.5 h, and finally at RT for 4 h). Ultra-thin sections (70 nm) were mounted on grids, contrast-enhanced with uranyl acetate and lead citrate, and examined with an EM900 electron microscope (Zeiss, Germany). Images were captured using a Slow-scan CCD-Camera (TRS, Germany).

## Supplementary information

Supplemental material

Reproducibility checklist

Original western blots

## Data Availability

All data are presented in the main manuscript or the supplementary file. Additional information will be provided by the corresponding author upon reasonable request.
