# Supplementary material for: Acidosis induces RIPK1-dependent death of glioblastoma stem cells via acid-sensing ion channel 1a
Source: Cell Death Dis. 2022 Aug 12;13(8):702. doi: 10.1038/s41419-022-05139-3 (PMC9374719; doi:10.1038/s41419-022-05139-3)
Supplement: Supplementary file 1 — Supplemental material [file 41419_2022_5139_MOESM1_ESM.pdf]

Supplementary Information

Acidosis induces RIPK1-dependent death of glioblastoma stem cells via acid-sensing ion channel 1a

Jan Clusmann[1], Klaus-Daniel Cortés Franco[1], David Alejandro Corredor Suárez[1], Istvan Katona[2], Maria Girbes Minguez[1], Nina Boersch[1], Karolos-Philippos Pissas[1], Jakob Vanek[1], Yuemin Tian[1], Stefan Gründer[1]

[1]*Institute of Physiology, RWTH Aachen University, Aachen, Germany*
[2]*Institute of Neuropathology, RWTH Aachen University, Aachen, Germany*

Contents

Supplementary Fig. 1. Acidic pH does not affect the cell cycle of R8 cells. ……..………….. 2
Supplementary Fig. 2. Nec-1 does not affect the cell cycle of R54 and R8 cells. …………... 3
Supplementary Fig. 3. Sphere size is not affected by inhibitors of cell death or of ASIC1a..... 4
Supplementary Fig. 4. Nec-1 does not affect the proportion of viable and apoptotic R54 cells. ……………………………………………………………………………………… 5
Supplementary Fig. 5. Effects of MitTx and peptides resembling the ASIC1a C-terminus on sphere size. ……………………………………………………………………………...…6
Supplementary Fig. 6. mRNA expression and correlation with survival of ASICs, RIPK1, RIPK3 and MLKL in TCGA GBM data. ……..……………...7
Supplementary Table 1. Coding and amino acid sequences of ASIC1 wildtype and knockout. ……..…………….. 8
Supplementary Table 2. Coding and amino acid sequences of MLKL wildtype and knockouts. ……..…………….. 9

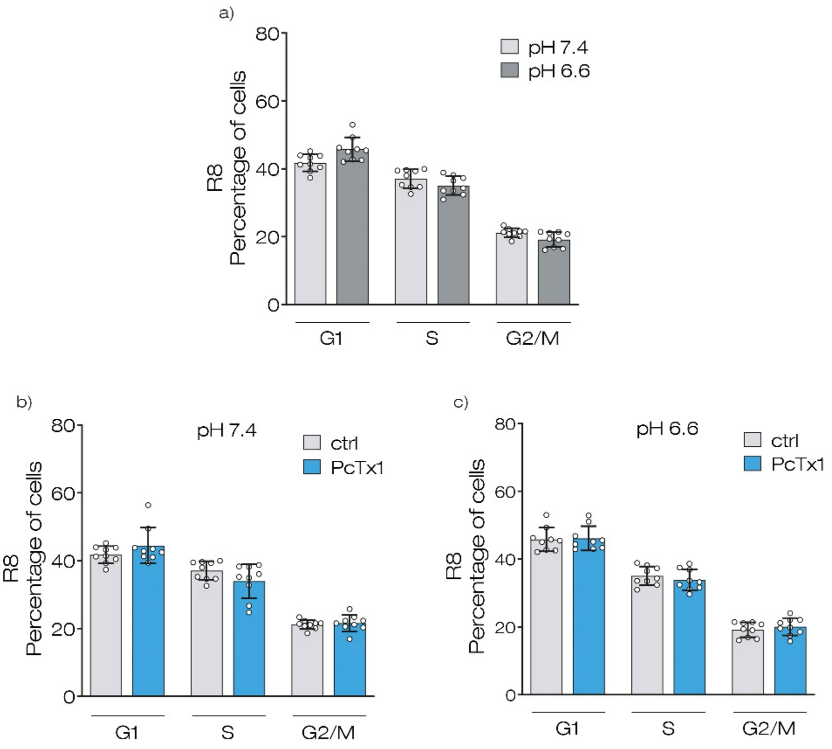

Supplementary Fig. 1. Acidic pH does not affect the cell cycle of R8 cells. (**a**) Percentage of R8 cells in different phases of the cell cycle at pH 7.4 and at pH 6.6. (**b**) Percentage of cells in different phases of the cell cycle at pH 7.4 without (DMSO-ctrl) and with PcTx1. (**c**) As in **b**, but for pH 6.6. Data for control cells in **b** and **c** are from panel **a**.

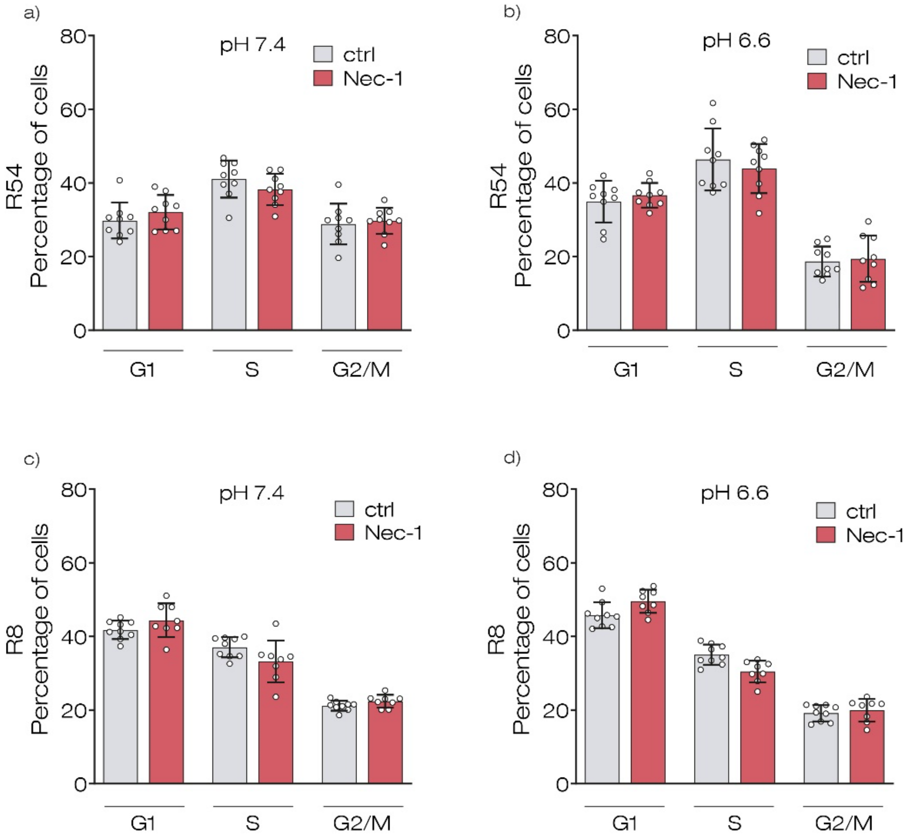

Supplementary Fig. 2. Nec-1 does not affect the cell cycle of R54 and R8 cells. (**a, b**) Percentage of R54 cells in different phases of the cell cycle at pH 7.4 or at pH 6.6, with or without 20 µM Nec-1. Data for ctrl cells are from Figure 1d, Supplementary Fig. 1a. Data are shown as mean ± SD of triplicate wells and are representative for three independent experiments. Data were compared by two-way ANOVA with Bonferroni correction. (**c, d**) As in **a** and **b**, but for R8 cells. Data for control cells are from Supplementary Fig. 1a.

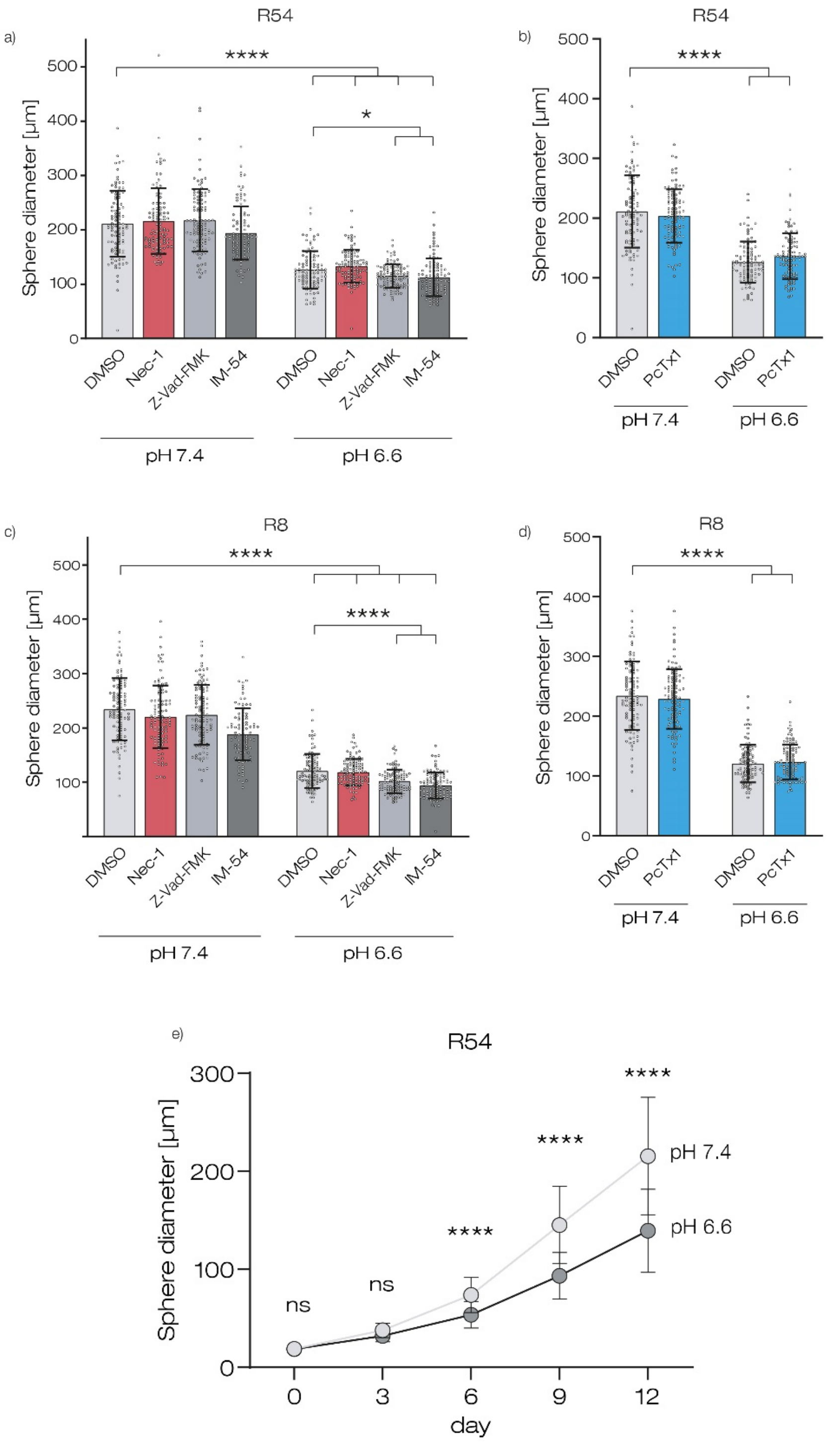

Supplementary Fig. 3. Sphere size is not affected by inhibitors of cell death or of ASIC1a. (**a**) Mean sphere diameters in µm of 100 spheres per condition from 2 biological replicates after 7-12 d at pH 7.4 or at pH 6.6 with 20 µM Nec-1, 20 µM Z-Vad FMK or 10 µM IM-54. Error bars represent SD. Data points represent single sphere diameters. * $p < 0.05$; **** $p < 0.0001$ (one-way ANOVA followed by Dunnett's post-hoc test). (**b**) as in **a**, but without and with 100 nM PcTx1. (**c**, **d**) as in **a**, **b**, but for R8 GSC. (**e**) Sphere diameter from d0 to d12 at pH 7.4 or pH 6.6. Mean and SD are shown. **** $p < 0.0001$ (two-way ANOVA followed by Sidak's multiple comparison test).

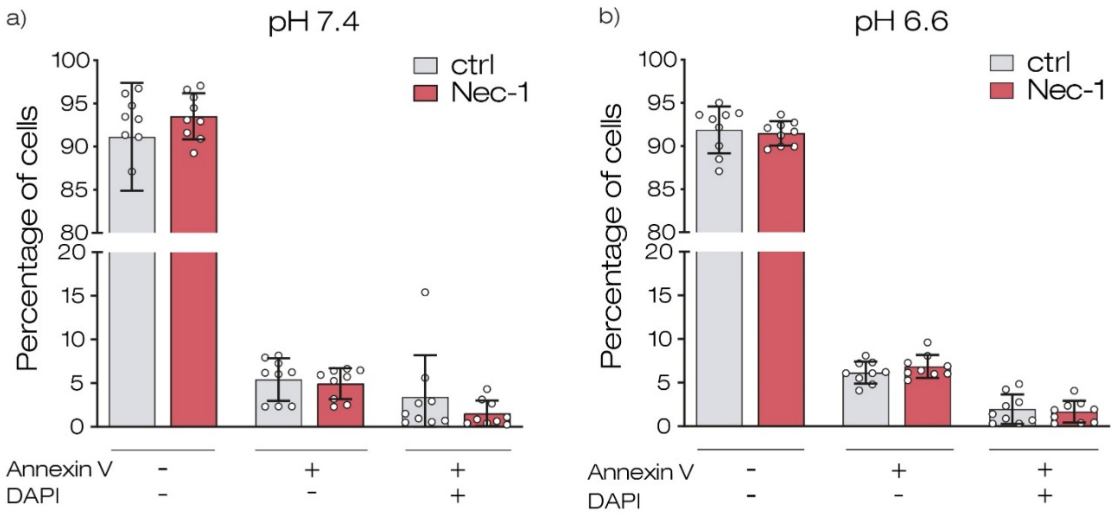

Supplementary Fig. 4. Nec-1 does not affect the proportion of viable and apoptotic R54 cells. (**a**) Percentage of viable, early apoptotic and late apoptotic R54 cells at pH 7.4 and DMSO (ctrl) or pH 7.4 and Nec-1. (**b**) as in **a** but for pH 6.6. Data are shown as mean ± SD of triplicate wells and are representative for three independent experiments. Data groups were compared by two-way ANOVA with Bonferroni correction.

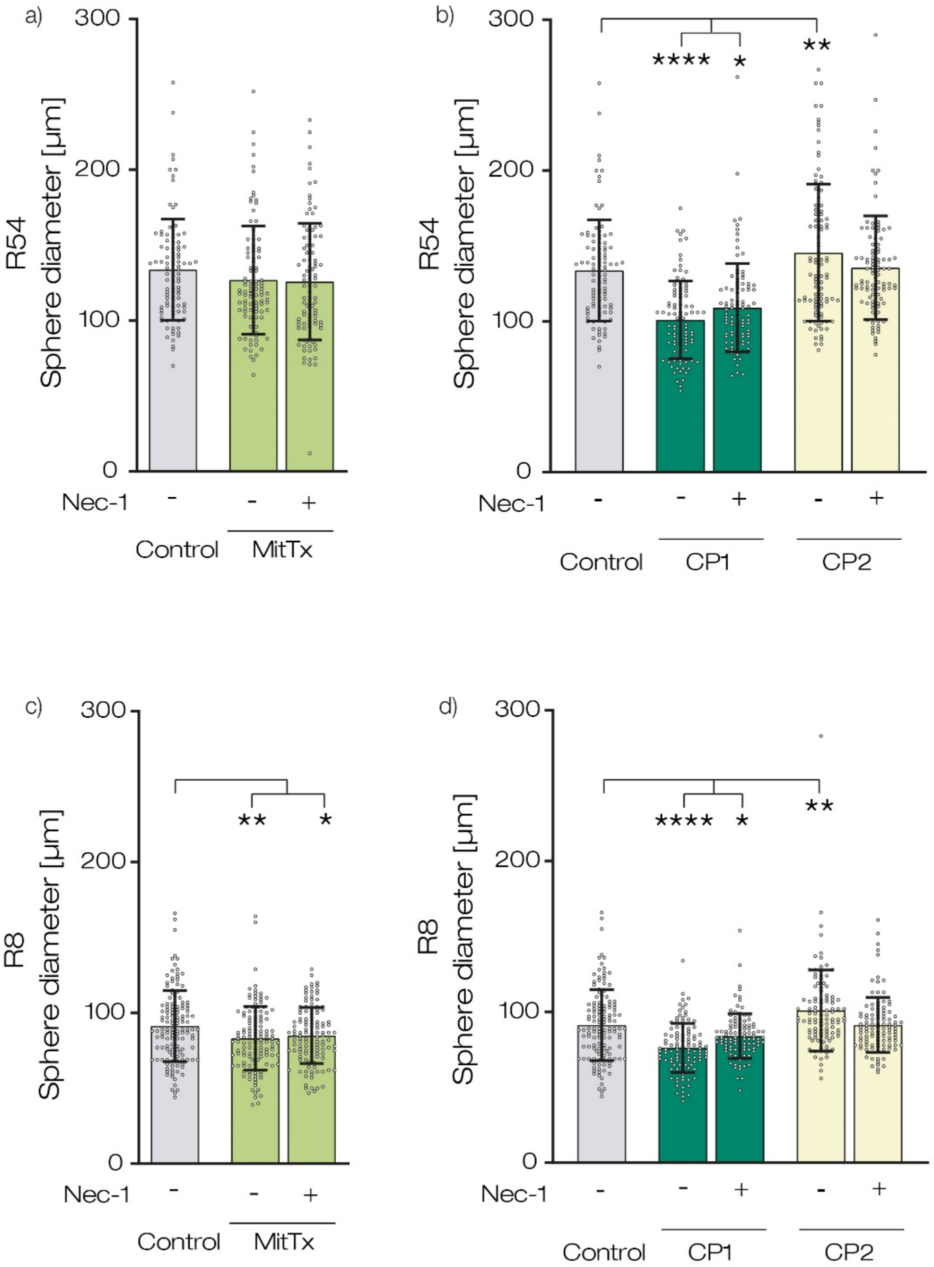

Supplementary Fig. 5. Effects of MitTx and peptides resembling the ASIC1a C-terminus on sphere size. (**a**) Mean sphere diameters in µm of 100 R54 spheres per condition from 2 biological replicates after 7 d at pH 7.4 with 20 nM MitTx with or without 20 µM Nec-1. (**b**) as in **a** but with 10 µM CP1 or 10 µM CP2. (**c, d**) as for **a** and **b** but for R8 cells. Error bars represent SD. Data points represent single sphere diameters. * $p < 0.05$; ** $p < 0.01$; **** $p < 0.0001$ (one-way ANOVA followed by Dunnett's post-hoc test).

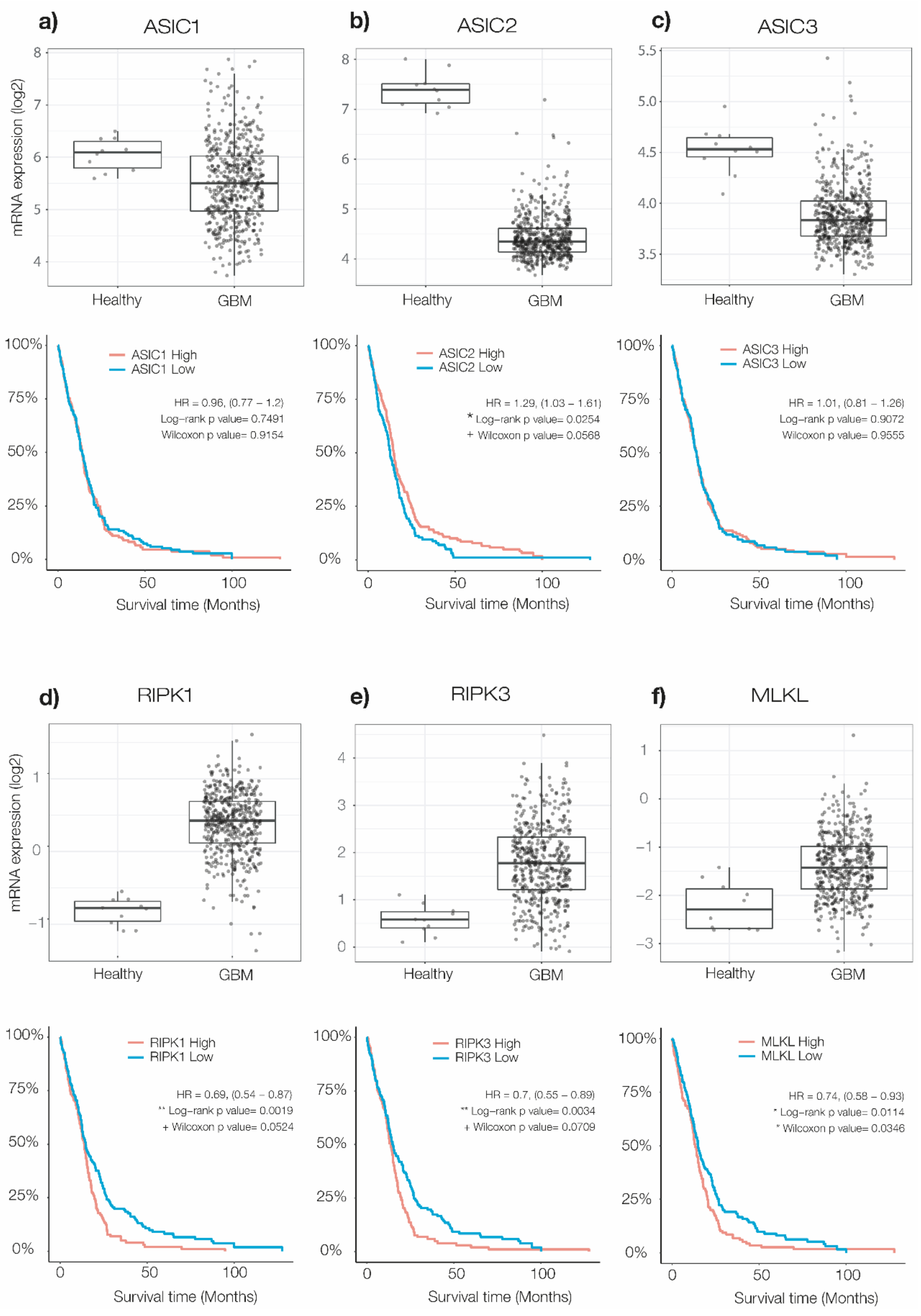

Supplementary Figure 6. mRNA expression and correlation with survival of ASICs, RIPK1, RIPK3 and MLKL in TCGA GBM data. (**a-c**, upper panel) mRNA expression levels from TCGA database (HG-U133A platform) in non-tumor vs GBM tissue (n=538 for GBM tissue) for ASIC1, ASIC2, ASIC3. (**a-c**, lower panel) Kaplan-Meier estimator survival analysis for IDH wt GBM in months (n=185-187) (**d-f**, upper panel) mRNA expression levels from TCGA database (Agilent-4502A platform) in non-tumor vs GBM tissue (n=499 for GBM tissue) for RIPK1, RIPK3 and MLKL. (**d-f**, lower panel) Kaplan-Meier estimator survival analysis for IDH wt GBM in months (n=167-172).

Supplementary Table 1. Coding and amino acid sequences of ASIC1 wildtype and knockout. Start and stop codons are underlined, the gRNA target is highlighted in green, the 17 bp deletion in red.

DNA sequence of ASIC1 WT	ATGGAACTGAAGGCCGAGGAGGAGGAGGTGGGTGGCGTCCAGCCGGTGAGCATCCAGGC<br>CTTCGCCAGCAGCTCCACACTGCACGGCCTGGCCCACATCTTCTCCTACGAGCGGCTGT<br>CTCTGAAGCGGGCACTGTGGGCCCTGTGCTTCCTGGGCTCCCTGGCTGTGCTGCTGTGT<br>GTGTGCACGGAGCGTGTGCAGTACTACTTCCACTACCACCATGTCACCAAGCTCGACGA<br>GGTGGCTGCCTCTCAGCTTACCTTCCCTGCTGTCACGCTGTGCAACCTCAACGAGTTCC<br>GCTTTAGCCAAGTCTCCAAGAATGACCTGTATCATGCTGGGGAGCTGCTGGCCCTGCTC<br>AACAACAGGTATGAGATACCAGACACACAGATGGCAGATGAAAAGCAGCTGGAGATACT<br>GCAGGACAAAGCCAACTTCCGCAGCTTCAAACCCAAACCCTTCAACATGCGTGAGTTCT<br>ACGACCGAGCTGGGCACGACATTCGAGACATGCTGCTCTCCTGCCACTTCCGGGGGGAG<br>GTCTGCAGCGCTGAAGACTTCAAGGTGGTCTTCACACGCTATGGAAAGTGCTACACGTT<br>CAACTCGGGCCGAGATGGGCGGCCGCGGCTGAAGACCATGAAGGGTGGGACGGGCAATG<br>GGCTGGAAATCATGCTGGACATCCAGCAGGACGAGTACCTGCCTGTGTGGGGGGAGACT<br>GACGAGACGTCCTTCGAAGCAGGCATCAAAGTGCAGATCCATAGTCAGGATGAACCTCC<br>TTTCATCGACCAGCTGGGCTTTGGCGTGGCCCCAGGCTTCCAGACCTTTGTGGCCTGCC<br>AGGAGCAGCGGCTCATCTACCTGCCCCCACCCTGGGGCACCTGCAAAGCTGTTACCATG<br>GACTCGGATTTGGATTTCTTCGACTCCTACAGCATCACTGCCTGCCGCATCGACTGTGA<br>GACGCGCTACCTGGTGGAGAACTGCAACTGCCGCATGGTGCACATGCCAGGGGATGCCC<br>CATACTGTACTCCAGAGCAGTACAAGGAGTGTGCAGATCCTGCTCTGGACTTCCTGGTG<br>GAGAAGGACCAGGAGTACTGCGTGTGTGAAATGCCTTGCAACCTGACCCGCTATGGCAA<br>AGAGCTGTCCATGGTCAAGATCCCCAGCAAAGCCTCAGCCAAGTACCTGGCCAAGAAGT<br>TCAACAAATCTGAGCAATACATAGGGGAGAACATCCTGGTGCTGGACATTTTCTTTGAA<br>GTCCTCAACTATGAGACCATTGAACAGAAGAAGGCCTATGAGATTGCAGGGCTCCTGGG<br>TGACATCGGGGGCCAGATGGGGCTGTTCATCGGGGCCAGCATCCTCACGGTGCTGGAGC<br>TCTTTGACTACGCCTACGAGGTCATTAAGCACAAGCTGTGCCGACGAGGAAAATGCCAG<br>AAGGAGGCCAAAAGGAGCAGTGCGGACAAGGGCGTGGCCCTCAGCCTGGACGACGTCAA<br>AAGACACAACCCGTGCGAGAGCCTTCGGGGCCACCCTGCCGGGATGACATACGCTGCCA<br>ACATCCTACCTCACCATCCGGCCCGAGGCACGTTCGAGGACTTTACCTGCTGA
DNA sequence of ASIC1 KO	ATGGAACTGAAGGCCGAGGAGGAGGAGGTGGGTGGCGTCCAGCCGGTGAGCATCCAGGC<br>CTTCGCCAGCAGCTCCACACTGCACGGCCTGGCCCACATCTTCTCCTACGAGCGGCTGT<br>CTCTGAAGCGGGCACTGTGGGCCCTGTGCTTCCTGGGCTCCCTGGCTGTGCTGCTGTGT<br>GTGTGCACGGAGCGTGTGCAGTACTACTTCCACTACCACCA~~TGTCACCAAGCTCGACG~~A<br>GGTGGCTGCCTCTCAGCTTACCTTCCCTGCTGTCACGCTGTGCAACCTCAACGAGTTCC<br>GCTTTAGCCAAGTCTCCAAGAATGACCTGTATCATGCTGGGGAGCTGCTGGCCCTGCTC<br>AACAACAGGTATGAGATACCAGACACACAGATGGCAGATGAAAAGCAGCTGGAGATACT<br>GCAGGACAAAGCCAACTTCCGCAGCTTCAAACCCAAACCCTTCAACATGCGTGAGTTCT<br>ACGACCGAGCTGGGCACGACATTCGAGACATGCTGCTCTCCTGCCACTTCCGGGGGGAG<br>GTCTGCAGCGCTGAAGACTTCAAGGTGGTCTTCACACGCTATGGAAAGTGCTACACGTT<br>CAACTCGGGCCGAGATGGGCGGCCGCGGCTGAAGACCATGAAGGGTGGGACGGGCAATG<br>GGCTGGAAATCATGCTGGACATCCAGCAGGACGAGTACCTGCCTGTGTGGGGGGAGACT<br>GACGAGACGTCCTTCGAAGCAGGCATCAAAGTGCAGATCCATAGTCAGGATGAACCTCC<br>TTTCATCGACCAGCTGGGCTTTGGCGTGGCCCCAGGCTTCCAGACCTTTGTGGCCTGCC<br>AGGAGCAGCGGCTCATCTACCTGCCCCCACCCTGGGGCACCTGCAAAGCTGTTACCATG<br>GACTCGGATTTGGATTTCTTCGACTCCTACAGCATCACTGCCTGCCGCATCGACTGTGA<br>GACGCGCTACCTGGTGGAGAACTGCAACTGCCGCATGGTGCACATGCCAGGGGATGCCC<br>CATACTGTACTCCAGAGCAGTACAAGGAGTGTGCAGATCCTGCTCTGGACTTCCTGGTG<br>GAGAAGGACCAGGAGTACTGCGTGTGTGAAATGCCTTGCAACCTGACCCGCTATGGCAA<br>AGAGCTGTCCATGGTCAAGATCCCCAGCAAAGCCTCAGCCAAGTACCTGGCCAAGAAGT<br>TCAACAAATCTGAGCAATACATAGGGGAGAACATCCTGGTGCTGGACATTTTCTTTGAA<br>GTCCTCAACTATGAGACCATTGAACAGAAGAAGGCCTATGAGATTGCAGGGCTCCTGGG<br>TGACATCGGGGGCCAGATGGGGCTGTTCATCGGGGCCAGCATCCTCACGGTGCTGGAGC<br>TCTTTGACTACGCCTACGAGGTCATTAAGCACAAGCTGTGCCGACGAGGAAAATGCCAG<br>AAGGAGGCCAAAAGGAGCAGTGCGGACAAGGGCGTGGCCCTCAGCCTGGACGACGTCAA

	AAGACACAACCCGTGCGAGAGCCTTCGGGGCCACCCTGCCGGGATGACATACGCTGCCA<br>ACATCCTACCTCACCATCCGGCCCGAGGCACGTTCGAGGACTTTACCTGCTGA
Amino acid sequence of ASIC1 WT	MELKAEEEEVGGVQPVSIQAFASSSTLHGLAHIFSYERLSLKRALWALCFLGSLAVLLC<br>VCTERVQYYFHYHHVTKLDEVAASQLTFPAVTLCNLNEFRFSQVSKNDLYHAGELLALL<br>NNRYEIPDTQMADEKQLEILQDKANFRSFKPKPFNMREFYDRAGHDIRDMLLSCHFRGE<br>VCSAEDFKVVFTRYGKCYTFNSGRDGRPRLKTMKGGTGNGLEIMLDIQQDEYLPVWGET<br>DETSFEAGIKVQIHSQDEPPFIDQLGFGVAPGFQTFVACQEQRLIYLPPPWGTCKAVTM<br>DSDLDFFDSYSITACRIDCETRYLVENCNCRMVHMPGDAPYCTPEQYKECADPALDFLV<br>EKDQEYCVCEMPCNLTRYGKELSMVKIPSKASAKYLAKKFNKSEQYIGENILVLDIFFE<br>VLNYETIEQKKAYEIAGLLGDIGGQMGLFIGASILTVLELFDYAYEVIKHKLCRRGKCQ<br>KEAKRSSADKGVALSLDDVKRHNPCESLRGHPAGMTYAANILPHHPARGTFEDFTC-
Amino acid sequence of ASIC1 KO	MELKAEEEEVGGVQPVSIQAFASSSTLHGLAHIFSYERLSLKRALWALCFLGSLAVLLC<br>VCTERVQYYFHYHQGGCLSAYLPCCHAVQPQRVPL-

Supplementary Table 2. Coding and amino acid sequences of MLKL wildtype and knockouts. Start and stop codons are underlined, the gRNA target is highlighted in green, deletions in red, and insertions in grey. Exon 4-8, which is not part of the CDS in MLKL isoform 1 (Zhao et al. 2012), is highlighted in turquoise; knockouts target both isoforms. KO1 induced a 134 bp insertion, which incidentally also carries a stop codon. KO2 induced a 106 bp insertion, also creating an early stop codon.

DNA sequence of MLKL WT	ATGGAAAATTTGAAGCATATTATCACCCTTGGCCAGGTCATCCACAAACGGTGTGAAGA<br>GATGAAATACTGCAAGAAACAGTGCCGGCGCCTGGGCCACCGCGTCCTCGGCCTGATCA<br>AGCCTCTGGAGATGCTCCAGGACCAAGGAAAGAGGAGCGTGCCCTCTGAGAAGTTAACC<br>ACAGCCATGAACCGCTTCAAGGCTGCCCTGGAGGAGGCTAATGGGGAGATAGAAAAGTT<br>CAGCAATAGATCCAATATCTGCAGGTTTCTAACAGCAAGCCAGGACAAAATACTCTTCA<br>AGGACGTGAACAGGAAGCTGAGTGATGTCTGGAAGGAGCTCTCGCTGTTACTTCAGGTT<br>GAGCAACGCATGCCTGTTTCACCCATAAGCCAAGGAGCGTCCTGGGCACAGGAAGATCA<br>GCAGGATGCAGACGAAGACAGGCGAGCTTTCCAGATGCTAAGAAGAGATAATGAAAAAA<br>TAGAAGCTTCACTGAGACGATTAGAAATCAACATGAAAGAAATCAAGGAAACTTTGAGG<br>CAGTATTTACCACCAAAATGCATGCAGGAGATCCCGCAAGAGCAAATCAAGGAGATCAA<br>GAAGGAGCAGCTTTCAGGATCCCCGTGGATTCTGCTAAGGGAAAATGAAGTCAGCACAC<br>TTTATAAAGGAGAATACCACAGAGCTCCAGTGGCCATAAAAGTATTCAAAAAACTCCAG<br>GCTGGCAGCATTGCAATAGTGAGGCAGACTTTCAATAAGGAGATCAAAACCATGAAGAA<br>ATTCGAATCTCCCAACATCCTGCGTATATTTGGGATTTGCATTGATGAAACAGTGACTC<br>CGCCTCAATTCTCCATTGTCATGGAGTACTGTGAACTCGGGACCCTGAGGGAGCTGTTG<br>GATAGGGAAAAAGACCTCACACTTGGCAAGCGCATGGTCCTAGTCCTGGGGGCAGCCCG<br>AGGCCTATACCGGCTACACCATTCAGAAGCACCTGAACTCCACGGAAAAATCAGAAGCT<br>CAAACTTCCTGGTAACTCAAGGCTACCAAGTGAAGCTTGCAGGATTTGAGTTGAGGAAA<br>ACACAGACTTCCATGAGTTTGGGAACTACGAGAGAAAAGACAGACAGAGTCAAATCTAC<br>AGCATATCTCTCACCTCAGGAACTGGAAGATGTATTTTATCAATATGATGTAAAGTCTG<br>AAATATACAGCTTTGGAATCGTCCTCTGGGAAATCGCCACTGGAGATATCCCGTTTCAA<br>GGCTGTAATTCTGAGAAGATCCGCAAGCTGGTGGCTGTGAAGCGGCAGCAGGAGCCACT<br>GGGTGAAGACTGCCCTTCAGAGCTGCGGGAGATCATTGATGAGTGCCGGGCCCATGATC<br>CCTCTGTGCGGCCCTCTGTGGATGAAATCTTAAAGAAACTCTCCACCTTTTCTAAGTAG

DNA sequence of MLKL KO1	ATGGAAAATTTGAAGCATATTATCACCCTTGGCCAGGTCATCCACAAACGGTGTGAAGA<br>GATGAAATACTGCAAGAAACAGTGCCGGCGCCTGGGCCACCGCGTCCTCGGCCTGATCA<br>AGCCTCTGGAGATGCTCCAGGACCAAGGAAAGAGGAGCGTGCCCTCTGAGAAGTTAACC<br>ACAGCCATGAACCGCTTCAAGGCTGCCCTGGAGGAGGCTAATGGGGAGATAGAAAAGTT<br>CAGCAATAGATCCAATATCTGCAGGTTTCTAACAGCAAGCCAGGACAAAATACTCTTCA<br>AGGACGTGGCACGAACCCCCCGTTCAGCCCGACCGCTGCGCCTTATCCGGTAACTATCG<br>TCTTGAGTCCAACCCGGTAAGACACGACTTATCGCCACTGGCAGCAGCCACAGGTAACA<br>GGATTAGCAGAGCGAGGTATGTAGAACAGGAAGCTGAGTGATGTCTGGAAGGAGCTCTC<br>GCTGTTACTTCAGGTTGAGCAACGCATGCCTGTTTCACCCATAAGCCAAGGAGCGTCCT<br>GGGCACAGGAAGATCAGCAGGATGCAGACGAAGACAGGCGAGCTTTCCAGATGCTAAGA<br>AGAGATAATGAAAAAATAGAAGCTTCACTGAGACGATTAGAAATCAACATGAAAGAAAT<br>CAAGGAAACTTTGAGGCAGTATTTACCACCAAAATGCATGCAGGAGATCCCGCAAGAGC<br>AAATCAAGGAGATCAAGAAGGAGCAGCTTTCAGGATCCCCGTGGATTCTGCTAAGGGAA<br>AATGAAGTCAGCACACTTTATAAAGGAGAATACCACAGAGCTCCAGTGGCCATAAAAGT<br>ATTCAAAAAACTCCAGGCTGGCAGCATTGCAATAGTGAGGCAGACTTTCAATAAGGAGA<br>TCAAAACCATGAAGAAATTCGAATCTCCCAACATCCTGCGTATATTTGGGATTTGCATT<br>GATGAAACAGTGACTCCGCCTCAATTCTCCATTGTCATGGAGTACTGTGAACTCGGGAC<br>CCTGAGGGAGCTGTTGGATAGGGAAAAAGACCTCACACTTGGCAAGCGCATGGTCCTAG<br>TCCTGGGGGCAGCCCGAGGCCTATACCGGCTACACCATTCAGAAGCACCTGAACTCCAC<br>GGAAAAATCAGAAGCTCAAACTTCCTGGTAACTCAAGGCTACCAAGTGAAGCTTGCAGG<br>ATTTGAGTTGAGGAAAACACAGACTTCCATGAGTTTGGGAACTACGAGAGAAAAGACAG<br>ACAGAGTCAAATCTACAGCATATCTCTCACCTCAGGAACTGGAAGATGTATTTTATCAA<br>TATGATGTAAAGTCTGAAATATACAGCTTTGGAATCGTCCTCTGGGAAATCGCCACTGG<br>AGATATCCCGTTTCAAGGCTGTAATTCTGAGAAGATCCGCAAGCTGGTGGCTGTGAAGC<br>GGCAGCAGGAGCCACTGGGTGAAGACTGCCCTTCAGAGCTGCGGGAGATCATTGATGAG<br>TGCCGGGCCCATGATCCCTCTGTGCGGCCCTCTGTGGATGAAATCTTAAAGAAACTCTC<br>CACCTTTTCTAAGTAG
DNA sequence of MLKL KO2	ATGGAAAATTTGAAGCATATTATCACGGGGGAGGGGCAAACAACAGATGGCTGGCAACT<br>AGAAGGCACAGTCGAGGCTGATCAGCGAGCTCTAGTTAGAATTCCTTGTA~~GC~~CAGCTCG<br>TCCATGCCGAGAGTGA~~T~~CATCCACAAACGGTGTGAAGAGATGAAATACTGCAAGAAACA<br>GTGCCGGCGCCTGGGCCACCGCGTCCTCGGCCTGATCAAGCCTCTGGAGATGCTCCAGG<br>ACCAAGGAAAGAGGAGCGTGCCCTCTGAGAAGTTAACCACAGCCATGAACCGCTTCAAG<br>GCTGCCCTGGAGGAGGCTAATGGGGAGATAGAAAAGTTCAGCAATAGATCCAATATCTG<br>CAGGTTTCTAACAGCAAGCCAGGACAAAATACTCTTCAAGGACGTGAACAGGAAGCTGA<br>GTGATGTCTGGAAGGAGCTCTCGCTGTTACTTCAGGTTGAGCAACGCATGCCTGTTTCA<br>CCCATAAGCCAAGGAGCGTCCTGGGCACAGGAAGATCAGCAGGATGCAGACGAAGACAG<br>GCGAGCTTTCCAGATGCTAAGAAGAGATAATGAAAAAATAGAAGCTTCACTGAGACGAT<br>TAGAAATCAACATGAAAGAAATCAAGGAAACTTTGAGGCAGTATTTACCACCAAAATGC<br>ATGCAGGAGATCCCGCAAGAGCAAATCAAGGAGATCAAGAAGGAGCAGCTTTCAGGATC<br>CCCGTGGATTCTGCTAAGGGAAAATGAAGTCAGCACACTTTATAAAGGAGAATACCACA<br>GAGCTCCAGTGGCCATAAAAGTATTCAAAAAACTCCAGGCTGGCAGCATTGCAATAGTG<br>AGGCAGACTTTCAATAAGGAGATCAAAACCATGAAGAAATTCGAATCTCCCAACATCCT<br>GCGTATATTTGGGATTTGCATTGATGAAACAGTGACTCCGCCTCAATTCTCCATTGTCA<br>TGGAGTACTGTGAACTCGGGACCCTGAGGGAGCTGTTGGATAGGGAAAAAGACCTCACA<br>CTTGGCAAGCGCATGGTCCTAGTCCTGGGGGCAGCCCGAGGCCTATACCGGCTACACCA<br>TTCAGAAGCACCTGAACTCCACGGAAAAATCAGAAGCTCAAACTTCCTGGTAACTCAAG<br>GCTACCAAGTGAAGCTTGCAGGATTTGAGTTGAGGAAAACACAGACTTCCATGAGTTTG<br>GGAACTACGAGAGAAAAGACAGACAGAGTCAAATCTACAGCATATCTCTCACCTCAGGA<br>ACTGGAAGATGTATTTTATCAATATGATGTAAAGTCTGAAATATACAGCTTTGGAATCG<br>TCCTCTGGGAAATCGCCACTGGAGATATCCCGTTTCAAGGCTGTAATTCTGAGAAGATC<br>CGCAAGCTGGTGGCTGTGAAGCGGCAGCAGGAGCCACTGGGTGAAGACTGCCCTTCAGA<br>GCTGCGGGAGATCATTGATGAGTGCCGGGCCCATGATCCCTCTGTGCGGCCCTCTGTGG<br>ATGAAATCTTAAAGAAACTCTCCACCTTTTCTAAGTAG

Amino acid sequence of MLKL WT	MENLKHIITLGQVIHKRCEEMKYCKKQCRRLGHRVLGLIKPLEMLQDQGKRSVPSEKLT<br>TAMNRFKAALEEANGEIEKFSNRSNICRFLTASQDKILFKDVNRKLSDVWKELSLLLQV<br>EQRMPVSPISQGASWAQEDQQDADEDRRAFQMLRRDNEKIEASLRRLEINMKEIKETLR<br>QYLPPKCMQEIPQEQIKEIKKEQLSGSPWILLRENEVSTLYKGEYHRAPVAIKVFKKLQ<br>AGSIAIVRQTFNKEIKTMKKFESPNILRIFGICIDETVTPPQFSIVMEYCELGTLRELL<br>DREKDLTLGKRMVLVLGAARGLYRLHHSEAPELHGKIRSSNFLVTQGYQVKLAGFELRK<br>TQTSMSLGTTREKTDRVKSTAYLSPQELEDVFYQYDVKSEIYSFGIVLWEIATGDIPFQ<br>GCNSEKIRKLVAVKRQQEPLGEDCPSELREIIDECRAHDPSVRPSVDEILKKLSTFSK-
Amino acid sequence of MLKL KO1	MENLKHIITLGQVIHKRCEEMKYCKKQCRRLGHRVLGLIKPLEMLQDQGKRSVPSEKLT<br>TAMNRFKAALEEANGEIEKFSNRSNICRFLTASQDKILFKDVARTPRSARPLRLIR-
Amino acid sequence of MLKL KO2	MENLKHIITGEGQTTDGWQLEGTVEADQRALVRIPCTARPCRE-

Zhao, Jie, et al. "Mixed lineage kinase domain-like is a key receptor interacting protein 3 downstream component of TNF-induced necrosis." Proceedings of the National Academy of Sciences 109.14 (2012): 5322-5327.
