# Supplementary figures and images for: Acidosis induces RIPK1-dependent death of glioblastoma stem cells via acid-sensing ion channel 1a
Source: Cell Death Dis. 2022 Aug 12;13(8):702. doi: 10.1038/s41419-022-05139-3 (PMC9374719; doi:10.1038/s41419-022-05139-3)

**Fig. 7a**

**pRIPK1**

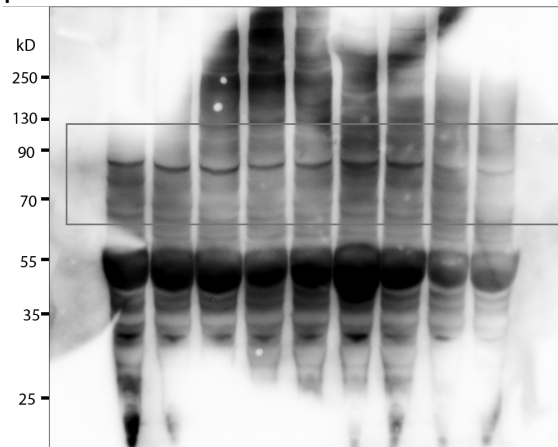

**RIPK1**

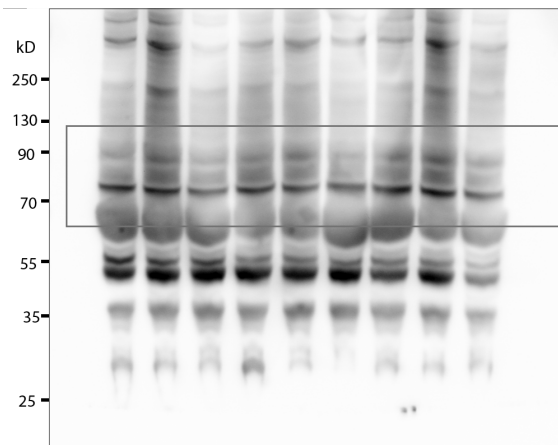

Supplement: Supplementary file 2 — Reproducibility checklist [file 41419_2022_5139_MOESM2_ESM.pdf]
